# Downregulation of PCYT2 by increased portal pressure safeguards liver regeneration after partial hepatectomy

**DOI:** 10.7150/thno.118755

**Published:** 2026-01-01

**Authors:** Guangyin Pu, Yayue Song, Qiushi Li, Jinjie Duan, Guangyan Wang, Wenjing Xiu, Jingwen Xu, Xiaoyu Zhao, Wenhui Dong, Tingting Lan, Rong Ai, Jingyi Zhang, Weiyan Sun, Deling Kong, Yi Zhu, Xu Zhang, Yang Liu, Chunjiong Wang

**Affiliations:** 1 Department of Physiology and Pathophysiology, the Province and Ministry Co-sponsored Collaborative Innovation Center for Medical Epigenetics, NHC Key Laboratory of Hormones and Development, Tianjin Medical University, Tianjin, China.; 2 Division of Neuroscience, Department of Brain Sciences, Imperial College London, London W12, 0NN, UK.; 3 State Key Laboratory of Medicinal Chemical Biology, Key Laboratory of Functional Polymer Materials of Ministry of Education, College of Chemistry, Nankai University, Tianjin, China.; 4 School of Public Health, Tianjin Medical University, Tianjin, China.; 5 Research Institute of Transplant Medicine, Tianjin First Central Hospital, School of Medicine; State Key Laboratory of Medicinal Chemical Biology, School of Life Science, Nankai University, Tianjin, China.; 6 Department of Biophysics, School of Basic Medical Sciences, Peking University Health Science Center, Beijing, China; 7 State Key Laboratory of Vascular Homeostasis and Remodeling, Peking University, Beijing, China.; 8 Tianjin Key Laboratory of Medical Epigenetics, Tianjin Medical University, Tianjin, China.

**Keywords:** liver regeneration, phospholipid remodeling, normothermic machine perfusion, nano-particle

## Abstract

**Rationale:** Metabolic remodeling occurs during partial hepatectomy (PHx)-induced liver regeneration. Phospholipid remodeling during this process and its subsequent impact on liver regeneration remain unknown. The remnant liver's ability to defend against injury is also essential for normal liver regeneration, although the underlying mechanisms remain unclear.

**Methods:** Phospholipidomics was performed to describe phospholipid remodeling after 70% PHx. Phosphate cytidylyltransferase 2, ethanolamine (PCYT2) was overexpressed in hepatocytes using adeno-associated virus under the thyroxine-binding globulin promoter. An *ex vivo* liver perfusion system was used to regulate portal pressure. GalNAc-conjugated PEG-PCL nano-particles (NPs) were developed to deliver the PCYT2 inhibitor, meclizine.

**Results:** We found a significant decrease in a series of phosphatidylethanolamine (PE) levels at 1 day after 70% PHx. PCYT2, an enzyme for PE synthesis, was downregulated by PHx. Higher portal pressure-induced shear stress is an early event after PHx. As a target gene of hepatocyte nuclear factor 4α, PCYT2 levels were decreased by higher portal pressure. Hepatocyte-specific PCYT2 overexpression aggravated liver damage after PHx by increasing reactive oxygen species levels, lipid peroxidation, and mitochondrial fragmentation. We observed higher hepatic PCYT2 levels in middle-aged mice than in young mice. PCYT2 inhibition by meclizine facilitates liver regeneration in middle-aged mice. Meclizine is also a blocker of the histamine H1 receptor, a membrane receptor. Therefore, we used NPs to deliver meclizine into cells to better target PCYT2 and prevent potential side effects. NP-meclizine improved liver regeneration in middle-aged mice, demonstrating higher therapeutic efficacy than carrier-free meclizine.

**Conclusions:** Decreased PCYT2 levels and PE content due to increased portal pressure protect hepatocytes from PHx-induced injury. Inhibiting PCYT2 with NP-meclizine promoted normal liver regeneration in middle-aged mice.

## Introduction

A normal liver has excellent regenerative capacity following major hepatectomy, with rapid recovery of its volume and function. Seventy percent of partial hepatectomy (PHx) induces liver regeneration without causing direct hepatocyte injury in the remaining lobes 1. Liver regeneration after PHx is closely related to the clinical situation, including hepatic resection surgery, liver transplantation, and two-stage hepatectomies 2, 3. The mechanisms underlying liver cell proliferation after 70% PHx have been well described but are not fully understood. After 70% PHx, the remnant liver's ability to defend against injury is also essential for normal liver regeneration 4. However, the underlying mechanisms remain unclear. Under conditions, including metabolic dysfunction-associated steatohepatitis (MASH) and aging, PHx leads to more severe liver injury 5-9. Therefore, investigating the mechanisms underlying hepatocyte protection may provide strategies to promote normal liver regeneration in these pathophysiological contexts.

Metabolic remodeling occurs during PHx-induced liver regeneration. Large amounts of lipids accumulate in the liver during the early phases 10. Gluconeogenesis is enhanced to overcome the low blood glucose levels induced by 70% PHx 11. Liver mitochondrial oxidative phosphorylation is inhibited, and glycolysis becomes the main source of ATP in the early phase of liver regeneration after 70% PHx 12, 13. Phospholipids are major components of the cellular membrane and generate bioactive signaling mediators that affect cellular functions 14. Five subclasses of phospholipids are defined based on their polar headgroups as follows: phosphatidylcholine (PC), phosphatidylethanolamine (PE), phosphatidylglycerol, phosphatidylinositol, and phosphatidylserine 14. Alterations in phospholipid components are closely associated with tissue physiology and pathophysiology. Three days after 70% PHx, monounsaturated PCs were correlated with hepatocyte proliferation 15. However, systematic research on phospholipid remodeling during liver regeneration after 70% PHx is lacking.

PE is the second most abundant multifunctional phospholipid in mammalian cells 16. The biogenesis and metabolism of PEs are linked to physiological and pathophysiological processes that regulate mitochondrial functions, membrane biological functions, protein functions, and lipid metabolism 17. The accumulation of PE and plasmalogen PE-truncated products also mediates ferroptosis 18. Specific changes in PE species during liver regeneration following PHx and their potential biological effects on regenerative processes are poorly understood. Moreover, it remains unclear whether the signals involved in initial liver regeneration are linked to PE metabolism.

In this study, we used phospholipidomics to describe phospholipid remodeling during liver regeneration after 70% PHx. We noted that a decrease in PE species was a feature of a change in phospholipid composition during the early stage of liver regeneration. Phosphate cytidylyltransferase 2, ethanolamine (PCYT2), an enzyme critical for PE biogenesis, was downregulated as a target gene of hepatocyte nuclear factor 4α (HNF4α). An isolated liver perfusion system was employed to explore the regulatory effects of increased portal pressure on HNF4α-PCYT2. We also overexpressed PCYT2 in hepatocytes and used the PCYT2 inhibitor meclizine *in vivo* and *in vitro* to elucidate the biological function of suppressed PE synthesis during the early stages of liver regeneration. We developed a nano-particle (NP) system to deliver meclizine and assessed its beneficial effects on liver regeneration in middle-aged mice.

## Materials and Methods

### Animals and treatments

Male C57BL/6J mice were obtained from SPF Biotechnology Co., Ltd. (Beijing). Eight-week-old wild-type male mice (C57BL/6J) were injected with AAV-PCYT2 (human)-flag bearing the TBG promoter (1 × 10^11^ viral genomes/mouse; GeneChem Co., Ltd.) to develop hepatocyte-specific PCYT2 overexpressing mice. Control mice were injected with control AAV. Ten days after AAV injection, the mice were subjected to 70% PHx. The mice were sacrificed at the indicated time points.

To investigate the effects of meclizine on liver regeneration, 8-week-old and 10-month-old wild-type male mice were intraperitoneally injected with the PCYT2 inhibitor meclizine (60 mg/kg; S1986, Selleck, Houston, TX) at 17 h and 3 h before surgery. The mice were sacrificed at the indicated time points.

To investigate the biodistribution and targetability of NP-meclizine in vivo, 8-week-old wild-type male mice (C57BL/6J) were intravenously injected with 80 μg Cy5-N-acetylgalactosamine (GalNAc)-NP-meclizine. NP-meclizine in various tissues was visualized using fluorescence imaging (IVIS SPECTRUM) at the indicated time points after intravenous injection.

To investigate the effects of NP-meclizine on liver regeneration, 10-month-old wild-type male mice were intravenously injected with 150 μg GalNAc-NP-meclizine (containing 25 μg meclizine) at 17 h and 6 h before the surgery. The mice were sacrificed at the indicated time points.

To examine the effects of NP-meclizine on acetaminophen (APAP)-induced liver injury, 8-week-old male mice were intravenously injected with 180 μg GalNAc-NP-meclizine (containing 25 μg meclizine) at 17 h and 6 h before they were intraperitoneal injected with APAP (300 mg/kg; HY-66005, MedChemExpress, NJ) for 24 h. Due to variations in the meclizine content across different NP batches, the NP-meclizine dose differed between APAP-treated and 70% PHx mice.

To develop MASH mouse model, 8-week-old male mice were fed with high fructose, palmitate, and cholesterol (FPC) diet for 24 weeks (Medicience, Yangzhou, China) 19.

All animal studies were performed in accordance with the Guide for the Care and Use of Laboratory Animals of the US National Institutes of Health (NIH Publication No. 85-23, updated 2011). This study was approved by the Laboratory Animal Management and Use Committee of the Tianjin Medical University (TMUaMEC2021049), Tianjin, China.

### The procedure of 70% PHx

PHx (70%) was performed as previously described 20. After the mice were anesthetized with isoflurane, the skin on the abdomen was disinfected. A midline incision of approximately 2-3 cm was made to expose the xiphoid process. The left and middle liver lobes were ligated and resected. After confirming the absence of bleeding, the abdominal cavity was closed by suturing.

### Perfusion of rat livers

Male Sprague-Dawley rats (225-250 g body weight) were obtained from SPF Biotechnology Co., Ltd. (Beijing). Rat livers were perfused with a small-animal isolated organ normothermic machine perfusion system, as previously reported 21-23. Rats were anesthetized with an intraperitoneal injection of sodium pentobarbital (60 mg/kg). Systemic heparinization was achieved by injecting 500 units of heparin into the tail vein. After opening the abdominal cavity, the hepatic arteries were isolated and ligated. The portal vein and bile duct were isolated. An indwelling needle syringe tube was inserted into the bile duct to drain the bile. The liver was connected to a small-animal isolated organ normothermic machine perfusion system through the portal vein. It has been reported that in the first 6 h after 70% PHx, the portal pressure increased approximately two-fold in rats 24. In our study, the whole liver or 30% of the liver (the liver was immediately isolated after 70% PHx) was perfused at an initial flow rate of 4 mL/min. Subsequently, the flow rate was adjusted to maintain a two-fold portal pressure in the 30% liver group (25-30 cmH_2_O vs. 10-14 cmH_2_O) for 6 h at 37 °C.

### Primary murine hepatocyte culture and treatment

Primary hepatocytes were isolated from the livers of 6-week-old male C57BL/6J mice as previously described 25. Briefly, 6-week-old mice were anesthetized, and the inferior vena cava was isolated. Then, the liver was perfused with 1 mL of sodium heparin solution (2 mg/mL), perfusate 1 (Krebs solution with 0.1 mM EGTA), and perfusate II (Krebs's solution with 2.74 mM CaCl_2_ and 0.05% collagenase type I). The isolated livers were filtered with precooled fetal bovine serum (FBS)-free RPMI 1640 culture solution through a 400 mesh filter. The hepatocyte suspension was centrifuged (50 × g for 2 min) and resuspended three times. Hepatocytes were cultured in RPMI 1640 medium supplemented with 10% FBS.

Hepatocytes (approximately 1×10^5^ cells per 3.5 cm dish) were infected with Ad-PCYT2 or Ad-Ctrl at 10 multiplicity of infection (MOI) for 48 h to induce PCYT2 overexpression. Cells were treated with or without 100 μM palmitate (PA) for another 24 h.

To inhibit PCYT2, hepatocytes were pretreated with meclizine (25 μM, S1986, Selleck, Houston, TX) or NP-meclizine for 17 h. Hepatocytes were then treated with or without 100 μM PA for another 24 h.

For PE treatment, hepatocytes were pretreated with PE (16:0/18:1; 10 μM, BD58707, Bidepharm, Shanghai) for 24 h; and were treated with or without 100 μM PA for another 24 h.

To knock down *Hnf4α*, the hepatocytes were treated with si-Hnf4α (Sc-35574, Santa Cruz Biotechnology, Dallas, TX) for 24 or 48 h. To overexpress *HNF4α*, the hepatocytes were transfected with Flag-tagged HNF4α (human)-expressing plasmids (Sangon Biotech, Shanghai) for 24 or 48 h. The HNF4α (human)-expressing plasmids were transfected with Lipofectamine®3000 (L3000-015, Invitrogen, Carlsbad, CA) according to the manufacturer's instructions.

For shear stress experiments, primary hepatocytes were cultured on glass slides and subjected to laminar shear stress of 4 dyn/cm² for 6 h using a flow system based on precision pump drive systems (Masterflex, USA) 26. Hepatocytes cultured under static conditions (0 dyn/cm²) were used as the control. The flow chamber was maintained at 37 °C and gassed with 95% humidified air and 5% CO₂.

### Cell culture and senescent cell model

EA.hy926 cells were sourced from the Cell Bank/Stem Cell Bank of the Chinese Academy of Sciences (Shanghai, China). HepG2 and RAW264.7 cells were obtained from the American Type Culture Collection. These cells were cultured in Dulbecco's modified Eagle's medium with 10% FBS. To induce senescence, HepG2 and RAW264.7 cells were treated with 2 μM Doxorubicin (DOX) hydrochloride (HY-15142, MedChemExpress, NJ) for 2 h, followed by a 4-day culture in DOX-free medium. EA.hy926 cells were treated with 0.5 μM DOX for 2 h and then cultured in DOX-free medium for 2 days 27, 28. Senescent cells were maintained in FBS-free medium for 24 h to collect conditioned medium, which was used to treat non-senescent HepG2 cells for 24 h.

### Sample preparation for liquid chromatography‒tandem mass spectrometry (LC‒MS/MS)

Ten milligrams of liver tissue or 5×10^5^ isolated hepatocytes were homogenized at 4 °C in 400 µL of 75% methanol (containing 0.01 M butylated hydroxytoluene) and 10 µL phospholipid internal standards (Avanti Research, Birmingham, AL). The mixture was centrifuged at 12,000 rpm for 15 min at 4 °C. Then, l mL of methyl tert-butyl ether was added, and the mixture was vortexed at room temperature for 30 min. Subsequently, 250 µL water was added, and the mixture was centrifuged at 12,000 rpm for 15 min at 4 °C after standing for 10 min at room temperature. The upper organic phase was transferred to a new tube and evaporated to dryness under a gentle stream of nitrogen. The sample was then reconstituted in 200 µL methanol. After vigorous mixing, the samples were filtered using a 0.22-μm centrifuge tube before loading into the mass spectrometer 29.

### LC-MS/MS for targeted lipidomics

A waters acquity UPLC liquid chromatograph was used, and mass spectrometry was performed on a QTrap 5500 (AB Sciex) triple quadrupole-ion trap mass spectrometer equipped with a UPLC BEH C18 column (1.7 μm, 100 × 2.1 mm i.d.). Glycerophospholipid metabolites were detected in both positive and negative ion modes. The column was maintained at a constant temperature of 25 °C. The mobile phase consisted of solvent A (60% acetonitrile) and solvent B [isopropanol/acetonitrile = 9:1 (containing 5 mM ammonium acetate)]. The mobile phase flow rate was maintained at 0.25 mL/min. The gradient elution profile was as follows: From 0 to 3 min, the proportion of solvent A was set at 75%. From 3 to 15 min, the proportion of solvent A was gradually reduced to 1% and remained constant for 2 min. From 17 to 19 min, the proportion of solvent A was gradually increased to 75% and remained constant for 1 min 30. Data were analyzed using the online tool MetaboAnalyst (www.metaboanalyst.ca/faces/ModuleView.xhtm).

### Synthesis of CH3O-polyethylene glycol (PEG)-block-Polycaprolactone (PCL) copolymer and BOC-NH-PEG-block-PCL copolymer

Block copolymers composed of PCL and PEG were synthesized 31, 32. First, ε-Caprolactone (A10299, Thermo Scientific, Waltham, MA) was polymerized via ring-opening polymerization, using mPEG [CH3O-PEG-OH (Yarebio, Shanghai) or BOC-NH-PEG-OH (PS2-OBC-2K-1g, Ponsure Biological, Shanghai)] and Sn(Oct)2 (S3252-100G, Sigma Aldrich, Darmstadt, Hesse) as the initiator and catalyst, respectively. mPEG (0.3 g, 0.15 mmol), ε-Caprolactone (1.365 g, 12.75 mmol), and two drops of Sn(Oct)2 were dissolved in dry toluene under an argon atmosphere. The reaction mixture was stirred at 110 °C for 24 h after three freeze-degassing‒melting cycles. The heating was stopped, and the reaction was terminated. The reaction mixture was added dropwise to cold diethyl ether, and the resulting polymer was obtained after 6 h of precipitation and then dried under reduced pressure at room temperature. The CH3O-PEG-block-PCL products were characterized by 1H-NMR in chloroform-D solvent.

### Synthesis of the NH2-PEG-block-PCL copolymer

A mixture of BOC-NH-PEG-block-PCL copolymer (1 g, 0.086 mmol), trifluoroacetic acid (5 mL), and dichloromethane (5 mL) was reacted at 25 °C for 24 h. Next, the reaction mixture was spun-dried, and anhydrous methanol was added to remove trifluoroacetic acid via a rotary evaporator. The crude product was dissolved in 1 mL of dimethyl sulfoxide (DMSO) and 100 µL of triethylamine and reacted at 25 °C for 4 h. Finally, the mixture was added dropwise to cold diethyl ether, and the resulting polymer was obtained after 6 h of precipitation and dried under reduced pressure at room temperature.

### Synthesis of the Cy5-PEG-block-PCL copolymer

The mixture of NH_2_-PEG-block-PCL copolymer (0.05 g, 0.0043 mmol), Cy5-NHS (0.0075 g, 0.012 mmol), and 4 mL of DMSO was reacted at 25 °C for 24 h. After the reaction, the mixture was transferred to a dialysis bag (molecular cutoff: 5 kDa) for 48 h, and the product was obtained by freeze-drying.

### Synthesis of the GalNAc-PEG-block-PCL copolymer

NH_2_-PEG-block-PCL copolymer (0.1 g, 0.0086 mmol), CDM-NAG (4.466 mg, 0.0094 mmol) (BQS336460-10mg, Gersion, Beijing), and 1.3 µL of triethylamine were dissolved in 9 mL of DMSO and reacted at 25 °C for 24 h in the dark. After the reaction, the solution was transferred to a dialysis bag (molecular cutoff: 5 kDa) for 48 h, and the product was obtained by freeze-drying. The GalNAc-PEG-block-PCL products were characterized by 1H-NMR in chloroform-D solvent.

### Preparation and characterization of GalNAc-NP-meclizine

Meclizine-loaded micelles were prepared using a dialysis method. CH3O-PEG-block-PCL copolymer, GalNAc-PEG-block-PCL copolymer, and Cy5-PEG-block-PCL copolymer were dissolved separately in tetrahydrofuran to obtain solutions of 5 mg/mL each. Meclizine was dissolved in tetrahydrofuran to obtain a 1 mg/mL solution. The GalNAc-NP-meclizine was prepared from a mixture of CH3O-PEG-block-PCL (402 µL), GalNAc-PEG-Block-PCL Copolymer (268 µL), and meclizine (335 µL). The mixture was added dropwise under sonication to 7 mL of ddH_2_O, sonicated for 40 min, and transferred to PBS for dialysis for 3 days. The meclizine content of the GalNAc-NP-meclizine was determined via ultraviolet spectroscopy at 232 nm and calculated using a concentration standard curve. Physical characterization of GalNAc-NP-meclizine was performed using dynamic light scattering and transmission electron microscopy. Cy5-GalNAc-NP-meclizine was prepared by replacing the 15% CH3O-PEG-block-PCL copolymer in GalNAc-NP-meclizine with the Cy5-PEG-block-PCL copolymer.

### Cellular uptake of Cy5-labeled GalNAc-NP-meclizine

HepG2 cells or primary mouse hepatocytes were treated with 3.75 μg/mL Cy5-GalNAc-NP-meclizine for 6 h. Cellular uptake of Cy5-GalNAc-NP-meclizine in HepG2 cells was detected using flow cytometry. Data were obtained using a BD FACSVerse flow cytometer and analyzed utilizing the FlowJo V10 software. These cells were stained with 10 μg/mL Hoechst 33342 (C0030, Solarbio, Beijing) for 25 min at 37 °C after treatment to visualize the cellular uptake of NP-meclizine. Images were acquired using an Axio-Imager LSM-800 microscope.

### Mitochondrial morphology assay

Mitochondria of live cells were stained with 100 nM MitoTracker probe (M7512, Invitrogen, Carlsbad, CA) for 15 min at 37 °C. Images were acquired using an Olympus FV1200 microscope. Qualitative scoring of the mitochondrial network morphology was performed on 50 hepatocytes using a previously described grading system 33.

### Determination of intracellular reactive oxygen species (ROS) level

Intracellular ROS levels were measured using DCFH-DA (S0033S; Beyotime, Shanghai) according to the manufacturer's instructions. Specifically, processed hepatocytes were loaded with 10 µM DCFH-DA for 20 min before being washed with PBS. The hepatocytes were washed with PBS three times. Images were acquired using a Leica DFC3000 G microscope, and the relative fluorescence intensity was quantified using Image-Pro PLUS software (6.0.0.260).

### Western blotting

Protein was extracted from cultured hepatocytes or liver tissues via high-efficiency RIPA tissue cell rapid lysis buffer containing phenylmethylsulfonyl fluoride (R0010, Solarbio, Beijing) with protein phosphatase inhibitors (Applygen, Beijing). After separation via SDS‒polyacrylamide gel electrophoresis, the proteins were transferred to a polyvinylidene fluoride membrane. The membranes were incubated with primary antibodies, including anti-PCYT2 (14827-1-AP, Proteintech, Wuhan), anti-PCNA (EPR3821, Abcam, Cambridge), anti-HNF4α (EPR16885, Abcam, Cambridge), anti-IL6 (AF0201, Beyotime, Shanghai), anti-EIF5 (sc-28309, Santa Cruz, Dallas, TX), or anti-β-Actin (66009-1-Ig, Proteintech, Wuhan) antibodies overnight at 4 °C, followed by incubation with secondary antibodies for 1 h at room temperature. The immunoblots were detected through enhanced chemiluminescence (Sigma Aldrich, Darmstadt, Hesse).

### RNA extraction and quantitative RT‒PCR

Total RNA was extracted from cultured hepatocytes or liver tissues with TRIzol (Transgene, Beijing) according to the manufacturer's instructions and subsequently reverse transcribed to cDNA via the RevertAid First Strand cDNA Synthesis Kit (K1622; Thermo Scientific, Waltham, MA) for PCR. The primer sequences are provided in Table S1.

### Histological analyses

For hematoxylin-eosin (H&E) staining and Oil Red O staining were performed as previously described 27. For immunohistochemical staining, paraffin sections were incubated with anti-PCYT2 (14827-1-AP, Proteintech, Wuhan, 1:200 dilution) and anti-Ki67 (ab16667, Abcam, Cambridge, 1:200 dilution) antibodies overnight at 4 °C and with secondary antibodies (A0208, Beyotime, Shanghai, 1:200 dilution) for 1 h at room temperature. The reaction was developed with diaminobenzidine staining.

### Determination of liver and plasma triglyceride (TG) levels

Hepatic lipids were extracted as previously described 27. The TG levels in the liver and plasma were determined using a TG determination kit (BioSino Bio-Technology & Science, Beijing).

### Malondialdehyde (MDA) detection

MDA was measured via an MDA Assay Kit (Nanjing Jiancheng Bioengineering Institute, Nanjing) according to the manufacturer's instructions.

### Alanine aminotransferase (ALT) detection

Plasma ALT levels were measured using an ALT Assay Kit (Nanjing Jiancheng Bioengineering Institute, Nanjing) according to the manufacturer's instructions.

### Statistical analysis

GraphPad Prism version 9.0.0 (GraphPad Software Inc., San Diego, CA) was used for all the statistical analyses. The data are presented as the mean ± SEM. We tested the normality of the data via the Shapiro‒Wilk normality test. For normally distributed data, two or more groups were compared via Student's t test or one-way ANOVA. For nonnormally distributed data, comparisons between two groups were performed via the Mann‒Whitney test, and comparisons among three or more groups were performed via the Kruskal‒Wallis test. The survival rate was analyzed via the log-rank test. Statistical significance was set at *p* < 0.05.

## Results

### The PE content substantially decreased at 1 day after 70% PHx

**Figure 1:**
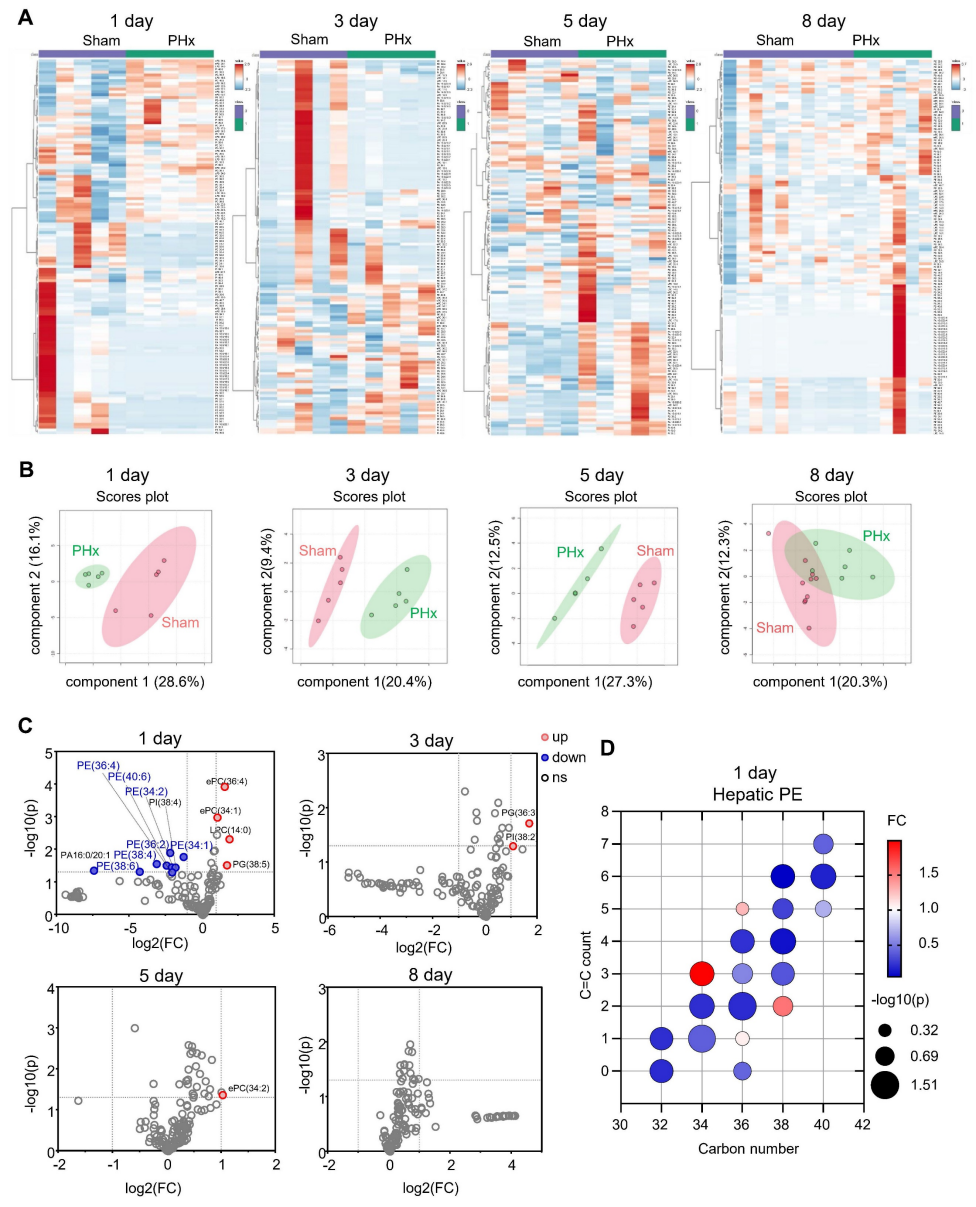
** The changes in phospholipid contents in mouse livers during liver regeneration after 70% partial hepatectomy (PHx).** Eight-week-old mice underwent 70% PHx or sham surgery. Liver tissues were collected at 1, 3, 5, and 8 days postoperatively for lipidomic analysis. **(A)** Heatmap of liver phospholipid levels. **(B)** Sparse partial least squares-discriminant analysis (sPLS-DA) score plot. **(C)** Volcano plot of significantly changed phospholipids. **(D)** Bubble diagram of phosphatidylethanolamine (PE) levels in the liver at 1 day. n = 5-10 mice per group. ePC/PE: ether PC/PE.

To investigate the remodeling of liver phospholipids during liver regeneration after PHx, we collected mouse liver tissues at 1, 3, 5, and 8 days after 70% PHx and performed phospholipidomics (Figure 1A). Compared with the sham group, the features of liver phospholipid composition were distinct at 1, 3, and 5 days postoperatively, as evidenced by the heatmap of phospholipid content and sparse partial least squares-discriminant analysis (Figure 1B). However, 8 days postoperatively, the phospholipid composition was comparable between the two groups (Figure 1B). We found that a series of PEs [PE(38:6); PE(38:4); PE(36:4); PE(40:6); PE(36:2); PE(34:2); and PE(34:1)] significantly decreased 1 day after 70% PHx (Figure 1C-D), which is a characteristic change in phospholipid composition at the early stage of liver regeneration after PHx.

### PCYT2, an enzyme involved in PE synthesis, was downregulated by 70% PHx

**Figure 2:**
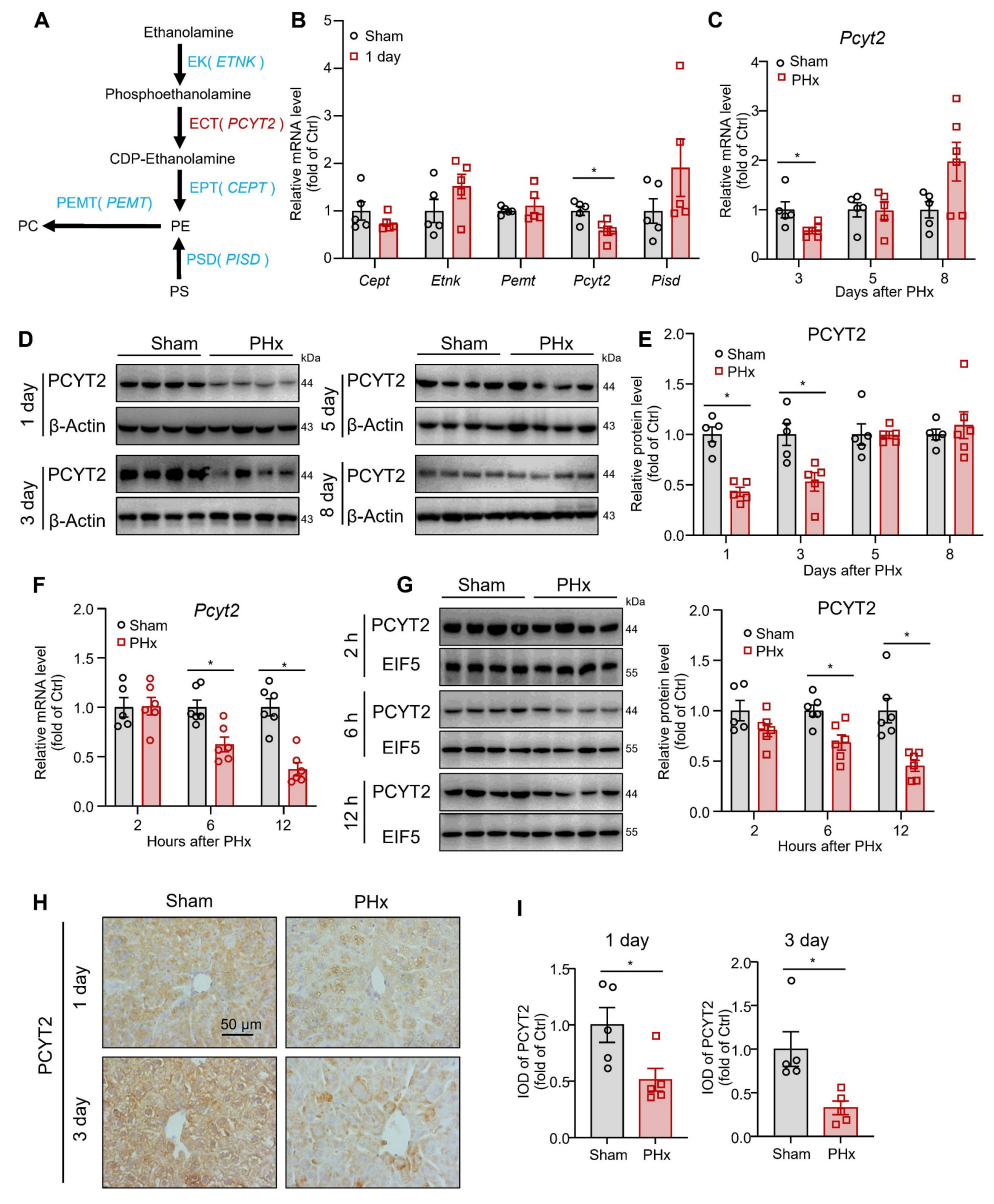
** PCYT2 was significantly decreased during early liver regeneration after 70% partial hepatectomy (PHx).** Eight-week-old mice underwent 70% PHx or sham surgery. Liver tissues were collected at the indicated time points. **(A)** Pathways of PE biogenesis and PE-PC conversion in mammalian cells. **(B)** mRNA levels of important enzymes involved in PE biogenesis and PE-PC conversion in the livers of mice 1 day postoperatively. **(C-G)** RNA and protein levels of PCYT2 in the livers of mice at the indicated time points postoperatively. β-Actin or EIF5 was used as the internal control. **(H-I)** Immunohistochemical staining of PCYT2 in liver sections of mice at 1 and 3 days postoperatively (scale bar = 50 μm). n = 5-6 mice per group. Data present the mean ± SEM. ∗*p <* 0.05.

In mammalian cells, PE is synthesized via CDP-ethanolamine and phosphatidylserine decarboxylase pathways. PE is converted to PC by phosphatidylethanolamine N-methyltransferase (PEMT; Figure 2A) 34. We tested the hepatic mRNA levels of these key enzymes for PE biogenesis and PE-PC conversion at 1 day after 70% PHx. The mRNA levels of *Pcyt2* decreased, whereas those of *Cept*, *Etnk*, *Pemt,* and *Pisd* remained unchanged (Figure 2B). PCYT2 converts cytidine triphosphate and phosphoethanolamine to CDP-ethanolamine and pyrophosphate and is the rate-limiting enzyme of the CDP-ethanolamine pathway 34. We noted that both the mRNA and protein levels of PCYT2 decreased at 1 and 3 days after 70% PHx but returned to normal at 5 days postoperatively (Figure 2B-E). To more accurately determine when PCYT2 started to decrease, we collected liver tissue samples at 2, 6, and 12 h after 70% PHx. We found that PCYT2 levels began to decline as early as 6 h postoperatively (Figure 2F-G). By searching the single-cell sequencing database Tabula Muris, we found that hepatocytes were the main cell type expressing *Pcyt2* in the mouse liver (Figure S1A). We isolated hepatocytes and non-parenchymal cells from mouse livers and demonstrated that PCYT2 was predominantly expressed in hepatocytes at the protein level (Figure S1B). Consistently, immunohistochemical staining revealed lower PCYT2 levels in hepatocytes from mouse livers at 1 and 3 days after 70% PHx (Figure 2H-I). We also isolated hepatocytes from mouse liver 1 day after 70% PHx and found decreased PE levels within these cells (Figure S1C).

### PCYT2 is a target gene of HNF4α, and its protein level was reduced by elevated portal pressure

**Figure 3:**
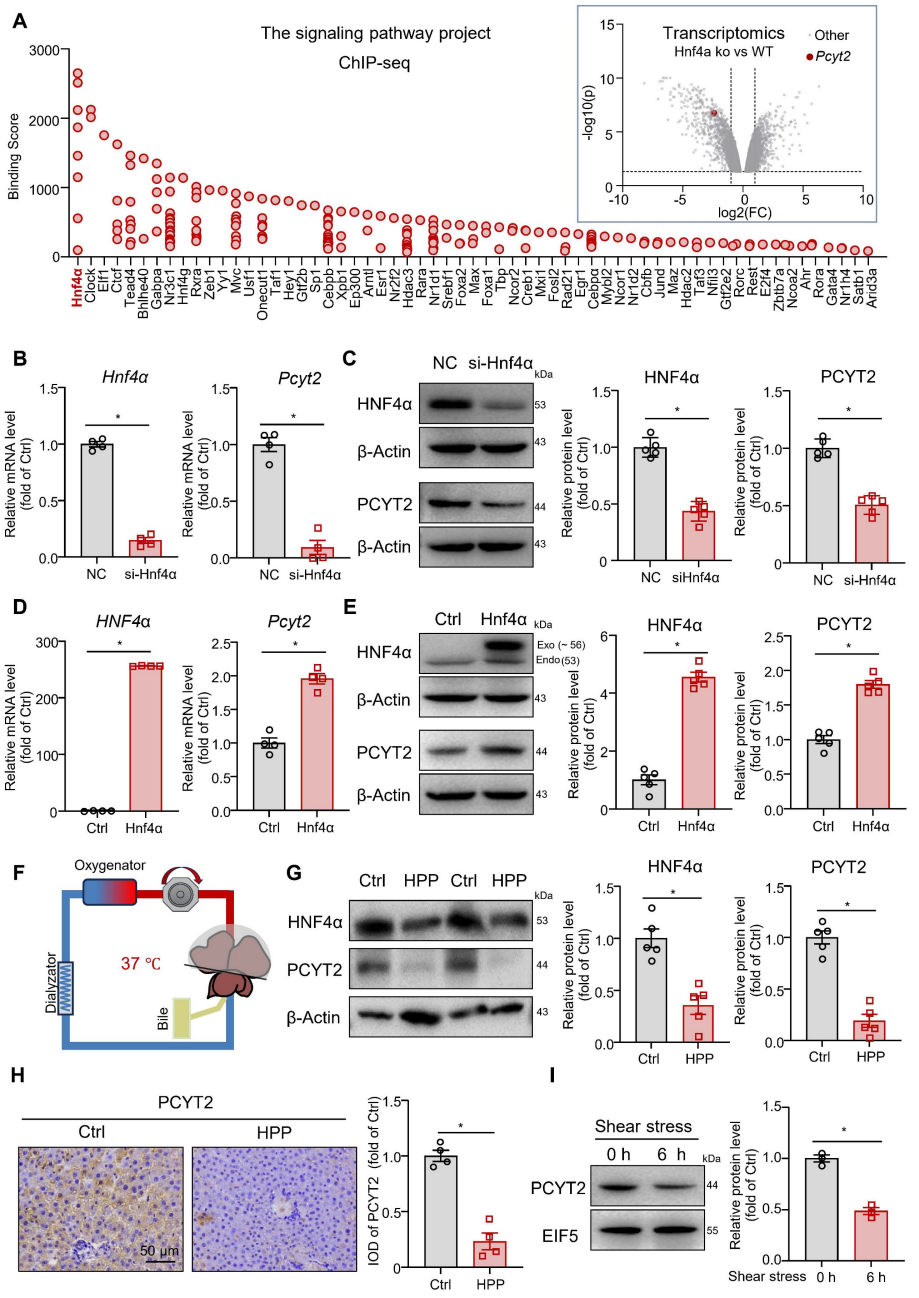
***Pcyt2* was a target gene of HNF4α, and both of them were decreased by higher portal pressure. (A)** The ChIP-sequencing database of the Signaling Pathways Project revealed the proteins binding with genome regions ± 10 kb from the transcriptional start site of the *Pcyt2* gene; the transcriptomics data from the Signaling Pathways Project showed that the *Pcyt2* mRNA levels were downregulated in *Hnf4α* knockout mice livers (Dataset DOI: 10.1621/Dhhr3ZavzL). **(B)** Primary hepatocytes were transfected with siHNF4α or the negative control for 24 h. The mRNA levels of *Hnf4a* and *Pcyt2*. **(C)** Primary hepatocytes were transfected with siHnf4α or the negative control for 48 h. The protein levels of HNF4α and PCYT2; β-Actin was used as the internal control. **(D)** Primary hepatocytes were transfected with HNF4α-expressing plasmids or the vectors for 24 h. The mRNA levels of *HNF4a* and *Pcyt2*. **(E)** Primary hepatocytes were transfected with HNF4α-expressing plasmids or the vectors for 48 h. The protein levels of HNF4α and PCYT2; β-Actin was used as the internal control. (B-E) n = 4-5 independent experiments. **(F-H)** Rat livers, either the whole liver or 30% of the liver (isolated immediately after 70% PHx), were isolated and perfused via an *ex vivo* perfusion system for 6 h. The portal perfusion pressure was maintained at approximately 2-fold greater in 30% of the livers than in the whole liver (F); (G) Protein levels of HNF4α and PCYT2 in the livers; β-Actin was used as the internal control. (H) Immunohistochemical staining of PCYT2 in liver sections of perfused livers (scale bar = 50 μm). n = 4-5 animals per group. **(I)** Primary hepatocytes were subjected to laminar shear stress of 4 dyn/cm^2^ or 0 dyn/cm^2^ (static conditions) for 6 h. Protein levels of HNF4α and PCYT2; EIF5 was used as the internal control; n = 3 independent experiments. Data present the mean ± SEM. ∗*p <* 0.05. Exo, exogenous; Endo, endogenous; HPP, higher portal pressure.

We explored the regulatory mechanisms involved in PCYT2 downregulation during the early stages of liver regeneration after 70% PHx. As *Pcyt2* mRNA levels were downregulated, we searched for transcription factors that regulate *Pcyt2* expression using the Signaling Pathways Project 35, 36. A search of Chip-seq data from the liver or hepatocytes revealed that HNF4α has the highest binding score with genome regions ± 10 kb from the transcriptional start site of the *Pcyt2* gene. A search of the transcriptomic data from the Signaling Pathways Project revealed that *Pcyt2* expression in the mouse liver was downregulated by *Hnf4α* knockout (Figure 3A) 37. These data indicate that *Pcyt2* is a target gene of HNF4α. HNF4α protein level decreases shortly after 70% PHx and recovers to normal levels 2 days postoperatively 38. Subsequently, we knocked down *Hnf4α* with siRNA in primary mouse hepatocytes and found that the mRNA and protein level of PCYT2 was greatly decreased (Figure 3B-C). In contrast, *HNF4α* overexpression increased the mRNA and protein levels of PCYT2 in hepatocytes (Figure 3D-E).

Higher portal pressure-induced shear stress is an early event after 70% PHx 39. Mechanical signaling regulates HNF4α expression 40. Thus, we explored the effects of increased portal pressure on HNF4α and PCYT2 protein levels with an *ex vivo* liver perfusion system. We perfused isolated rat livers (Figure 3F), either the whole liver or 30% of the liver (isolated immediately after 70% PHx), with the same perfusion solution. Portal perfusion pressure was maintained at approximately 2-fold greater in 30% of the liver than in the whole liver. After perfusion for 6 h, higher portal pressure led to a marked decrease in HNF4α and PCYT2 protein levels (Figure 3G). Immunohistochemical staining of PCYT2 also revealed that the protein level in the liver decreased with increasing portal pressure (Figure 3H). To further demonstrate whether PCYT2 level can be regulated by shear stress *in vitro*, we treated primary hepatocytes with shear stress. Six hours later, the PCYT2 protein level was also markedly decreased (Figure 3I). These data indicate that increased portal pressure by 70% PHx reduced HNF4α and PCYT2 protein levels.

### PCYT2 overexpression aggravated liver damage after 70% PHx

**Figure 4:**
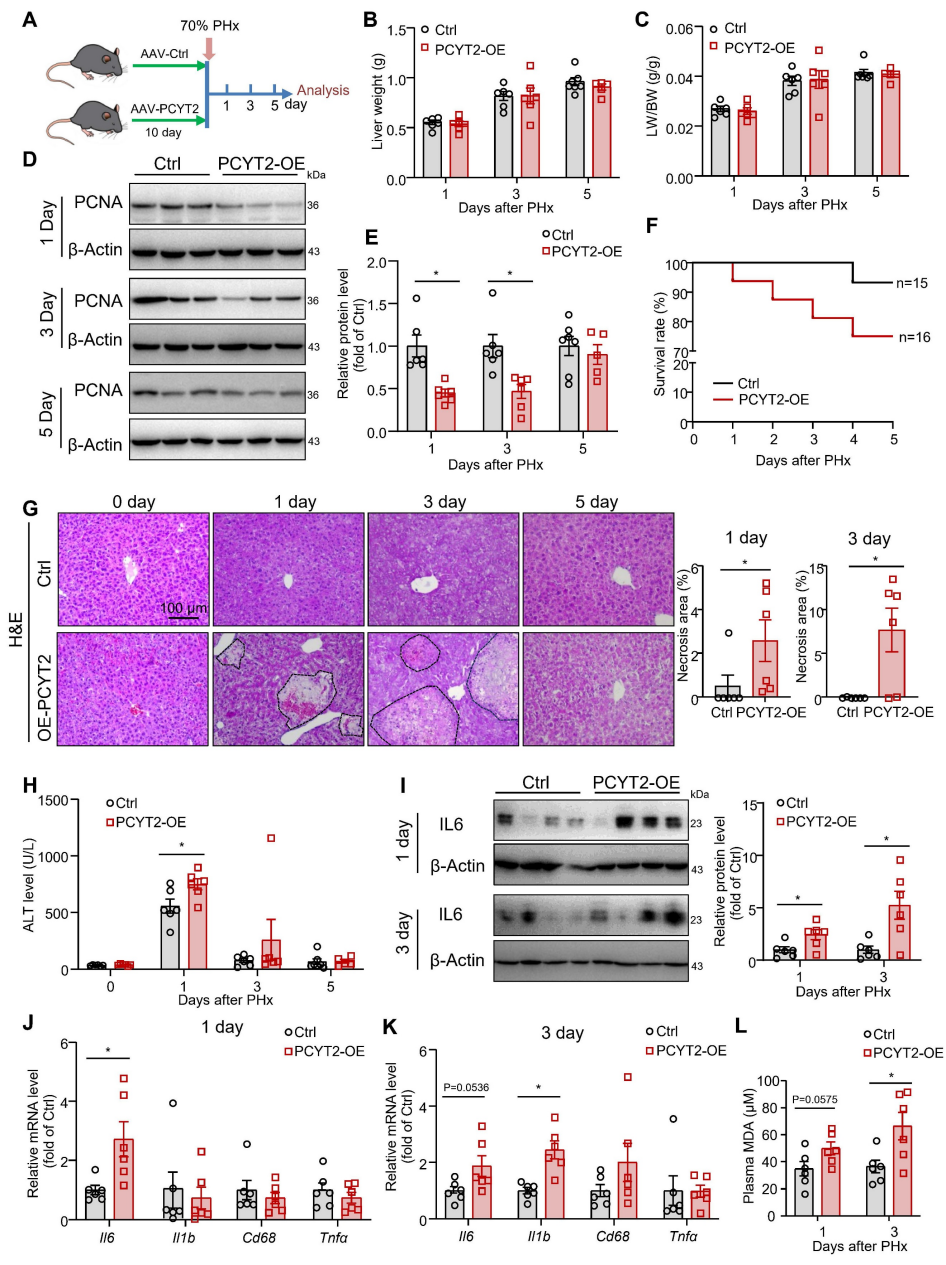
** Hepatocyte-specific PCYT2 overexpression exacerbated liver injury after 70% partial hepatectomy (PHx).** Eight-week-old mice were administered AAV-PCYT2-flags bearing the TBG promoter or control AAV through tail vein injection. After 10 days, the mice were subjected to 70% PHx, and the tissues were harvested for analysis at the indicated time points **(A)**. **(B-C)** Liver weights and liver-to-body weight ratios of mice. **(D-E)** Protein levels of PCNA in the liver; β-Actin was used as the internal control. **(F)** Survival rate of the mice after 70% PHx.** (G)** H&E staining of liver sections and quantitative analysis of liver necrosis areas (scale bar = 100 μm). **(H)** Plasma ALT levels.** (I)** Protein levels of IL-6 in the liver. β-Actin was used as the internal control. mRNA levels of inflammatory genes in the liver at 1 day **(J)** and 3 days **(K)** after 70% PHx. **(L)** Plasma MDA levels. n = 5-7 mice per group. Data present the mean ± SEM. ∗*p <* 0.05. LW/BW, liver-to-body weight ratio.

To demonstrate the impact of decreased PCYT2 level and PE content on liver regeneration, we used a TBG-promoter-controlled PCYT2-expressing AAV to overexpress PCYT2 in hepatocytes. Control and PCYT2-overexpressing mice were subjected to 70% PHx (Figure 4A and S1D). We found that liver weight, body weight, and liver/body weight ratio were not affected by PCYT2 overexpression at 1, 3, or 5 days after 70% PHx (Figure 4B-C, and S2A). However, PCYT2 overexpression decreased proliferating cell nuclear antigen (PCNA) levels (Figure 4D-E), and Ki67-positive cell numbers tended to decrease 3 days after 70% PHx (*p* = 0.0867; Figure S2B). These data demonstrate that PCYT2 overexpression might inhibit the proliferation pathway in the liver but is insufficient to affect the recovery of liver volume.

However, the survival rate after 70% PHx tended to decrease with PCYT2 overexpression (Figure 4F). Additionally, we detected more necrotic foci in mouse livers overexpressing PCYT2, at 1 and 3 days after 70% PHx (Figure 4G). In control mice, the plasma ALT level greatly increased 1 day after 70% PHx, and PCYT2 overexpression further increased the plasma ALT level at this time point (Figure 4H). After 70% PHx, large amounts of lipids accumulated in hepatocytes, and the levels of inflammatory cytokines substantially increased in the liver. Although these stress signals are important for driving hepatocyte proliferation, they can also cause hepatocyte damage 4, 41. Therefore, the liver should defend itself from injury during the early phases of PHx-induced liver regeneration. Our data indicate that decreased levels of PCYT2 are involved in the defense mechanisms. In support of this hypothesis, we found that the protein and mRNA levels of IL6 were higher in PCYT2 overexpressing mouse liver than in control mice, at 1 and 3 days after 70% PHx (Figure 4I**-**K). In control mice, inflammatory factor expression increased shortly after 70% PHx and returned to normal levels 3 days after PHx (Figure S3). Notably, the expression of inflammatory factors in the liver at 3 days after 70% PHx was increased by PCYT2 overexpression (Figure 4K). These data indicate that the proinflammatory phase was not resolved in the livers of PCYT2-overexpressing mice. Additionally, plasma MDA levels increased at 1 (*p* = 0.0575) and 3 days after 70% PHx (Figure 4L), suggesting increased lipid peroxidation. PCYT2 overexpression did not affect TG accumulation in the liver induced by 70% PHx, and plasma TG levels after 70% PHx remained unchanged (Figure S2C-D).

### ROS production, mitochondrial fragment, and lipid peroxidation were increased by PCYT2 overexpression and reduced by PCYT2 inhibition in hepatocytes challenged with free fatty acids

**Figure 5:**
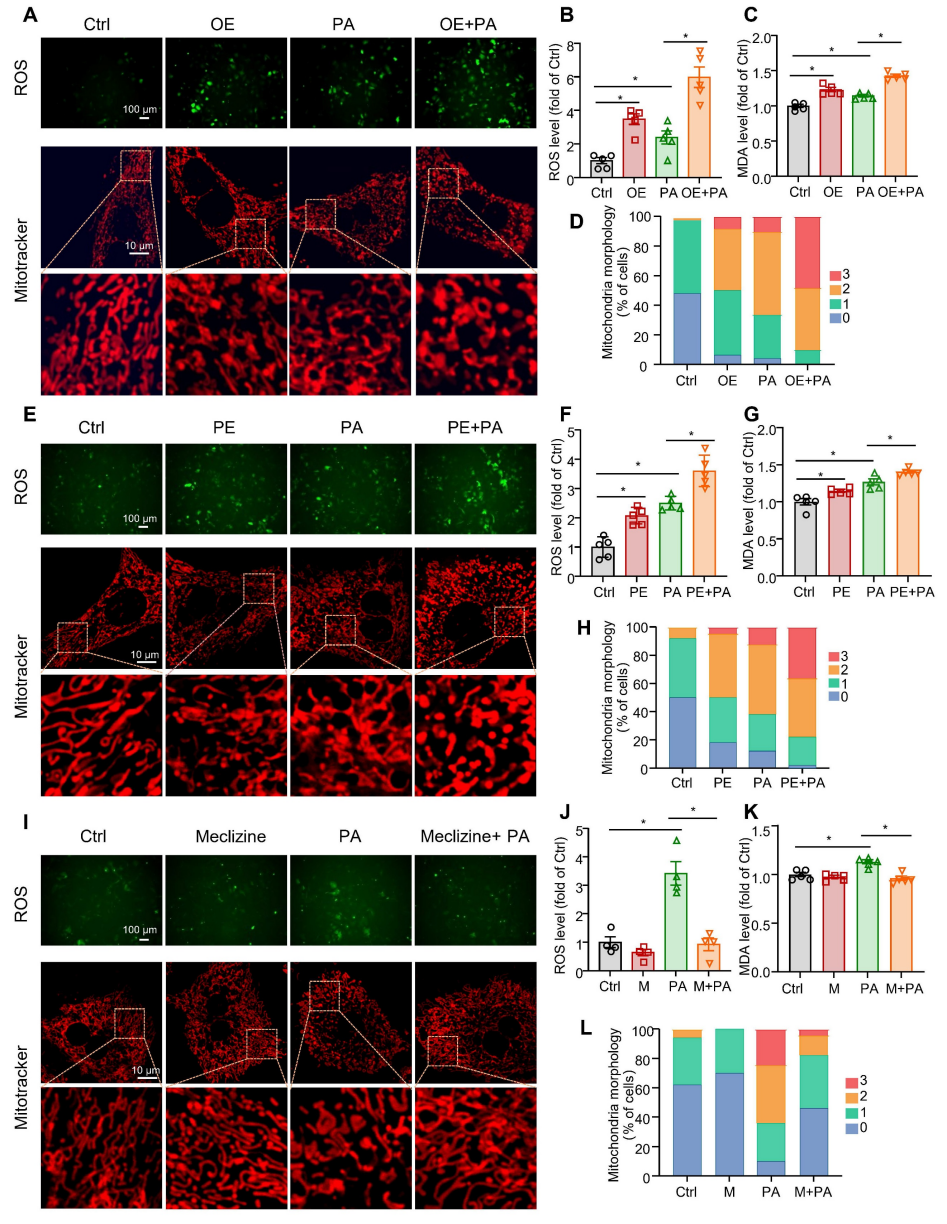
** The effects of overexpression and pharmacological inhibition of PCYT2 on ROS and MDA levels, and mitochondrial fragmentation in fatty acid-overloaded hepatocytes. (A-D)** Primary mouse hepatocytes were treated with Ad-PCYT2 or Ad-Ctrl for 48 h, then the cells were treated with or without palmitate (PA) (100 μM) for another 24 h. **(E-H)** Primary mouse hepatocytes were treated with phosphatidylethanolamine (PE; 10 μM) for 24 h; subsequently, the cells were co-treated with or without PA (100 μM) for another 24 h. **(I-L)** Primary mouse hepatocytes were treated with meclizine (25 μM) for 17 h; then, the cells were co-treated with or without PA (100 μM) for another 24 h. Representative images of ROS in hepatocytes (upper panel of A, E and I; scale bar = 100 μm) and fluorescence analysis of ROS (B, F, and J); the MDA levels in hepatocytes (C, G, and K); (lower panel of A, E, and I; scale bar = 10 μm) mitochondrial morphology was evaluated via MitoTracker. (D, H, and L) Quantitative scoring of mitochondrial morphology in 50 hepatocytes from five biologically independent experiments. n = 4-5 independent experiments. Data present the mean ± SEM. ∗*p <* 0.05. OE, overexpressing; M, meclizine; PA, palmitate; 0, elongated; 1, mildly fragmented; 2, moderately fragmented; 3, highly fragmented.

Meclizine is a PCYT2 inhibitor that shifts energy metabolism from mitochondrial respiration to glycolysis 42, 43. Meclizine pre-conditioning reportedly protects against kidney ischemia/reperfusion injury and cardiac and cerebral ischemic injuries 43, 44. Consistently, previous studies have reported that mitochondrial respiration is suppressed during the early phase of liver regeneration after 70% PHx, with glycolysis serving as the primary source of ATP 12, 13. Excess ROS, mainly generated by mitochondrial respiration, may cause lipid peroxidation and cell injury 45. Therefore, we overexpressed PCYT2 with adenovirus in primary mouse hepatocytes treated with PA to mimic lipid accumulation during the early phase of liver regeneration. We found that PCYT2 overexpression increased ROS levels in hepatocytes treated with or without PA (Figure 5A-B; Figure S4). High ROS levels can cause cell death by increasing lipid peroxidation 46. We observed that both PCYT2 overexpression and PA treatment increased the MDA levels, a marker of lipid peroxidation, in hepatocytes. PCYT2 overexpression further increased the MDA levels in PA-treated hepatocytes (Figure 5C). Next, we tested the effects of PCYT2 on mitochondrial morphology and found that its overexpression aggravated PA-induced mitochondrial fragmentation (Figure 5A, D). We further treated the hepatocytes with PE. Consistent with PCYT2 overexpression, PE treatment increased ROS levels, lipid peroxidation, and mitochondrial fragmentation in PA-treated hepatocytes (Figure 5E-H). In contrast, meclizine treatment attenuated ROS production, MDA levels, and mitochondrial fragmentation in PA-treated hepatocytes (Figure 5I-L).

### PCYT2 inhibition by meclizine facilitated liver regeneration in middle-aged mice

Since PCYT2 levels returned to normal 5 days after 70% PHx, genetically sustained inhibition of PCYT2 may lead to abnormal liver regeneration. Thus, to explore whether PCYT2 inhibition at the early phase of liver regeneration promotes liver regeneration, we pretreated mice (8-week-old) with the PCYT2 inhibitor meclizine (Figure S5A). We found that meclizine increased the liver weight and the liver/body weight ratio at 1 day after PHx (Figure S5B-D). PCNA levels and Ki67 positive cell counts in the liver after 70% PHx were also increased by meclizine (Figure S5E-F). Therefore, pretreatment with meclizine promoted 70% PHx-induced hepatocyte proliferation in young mice. However, neither the control nor the meclizine-treated mice presented with substantial necrotic foci in the liver (Figure S5G). Meclizine treatment did not alter the expression levels of inflammatory factors and plasma ALT (Figure S5H-I). This may be because, under normal conditions, mice can undergo liver regeneration normally after 70% PHx. Further PCYT2 inhibition did not exert any additional protective effects on the liver.

**Figure 6:**
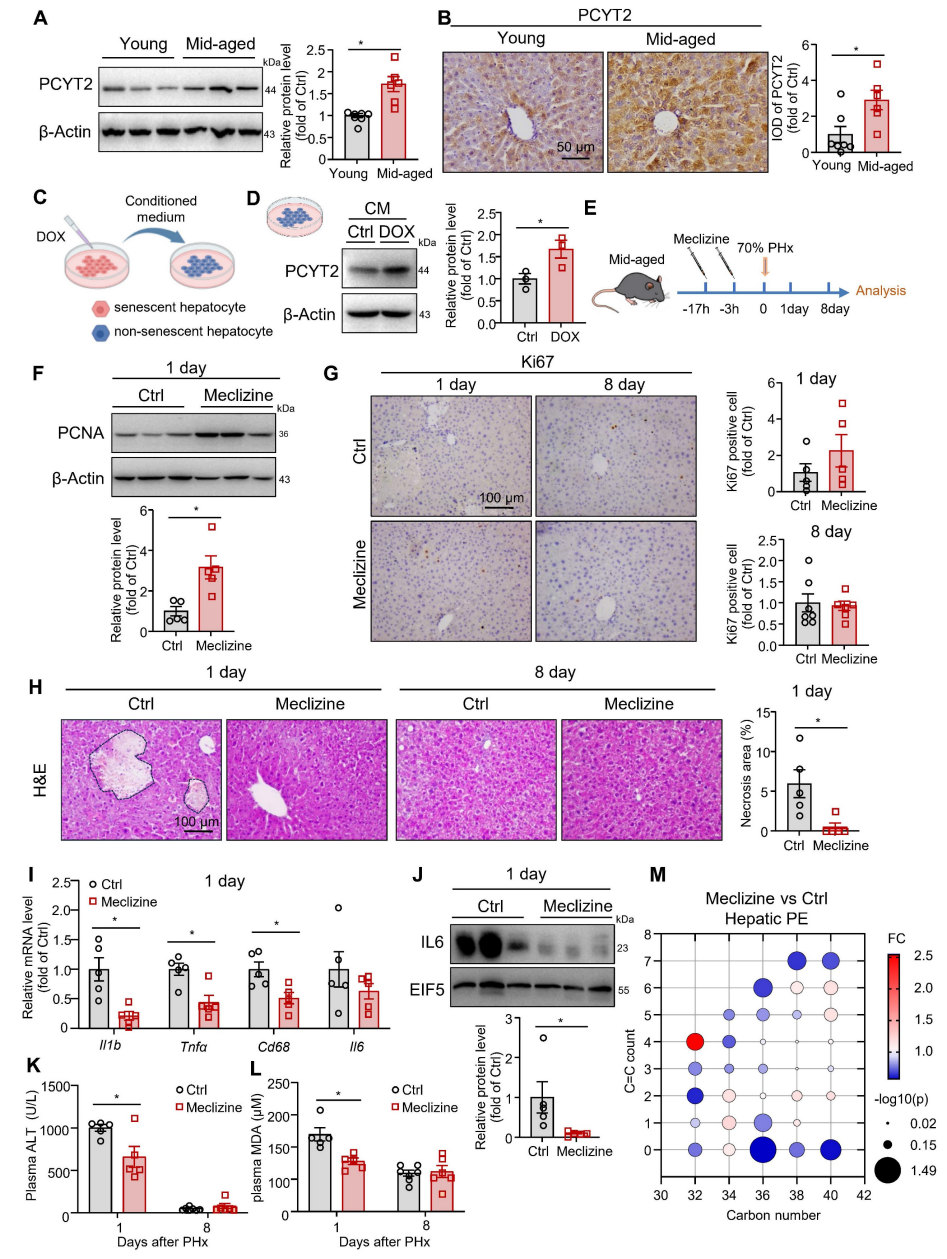
** Inhibition of PCYT2 by meclizine promoted normal liver regeneration in middle-aged mice. (A-B)** Protein levels and immunohistochemical staining (scale bar = 50 μm) of PCYT2 in the livers of 8-week-old and 12-month-old mice. β-Actin was used as the internal control. n = 6-7 mice per group. **(C)** HepG2 cells were treated with doxorubicin (DOX) to induce senescence. The conditioned medium was collected to treat non-senescent HepG2 cells. **(D)** The protein levels of PCYT2 in non-senescent hepatocytes treated with the conditioned medium; β-Actin was used as the internal control. n = 3 independent experiments. **(E-M)** Ten-month-old mice were administered 60 mg/kg meclizine or vehicle 17 h and 3 h before 70% PHx, and subsequently the tissues were harvested for analysis at 1 day (n = 5 mice per group) or 8 days (n = 6-7 mice per group) postoperatively (E). (F) Protein levels of PCNA in the liver of mice at 1 day postoperatively. β-Actin was used as the internal control. (G) Immunohistochemical staining of Ki67 in liver sections from mice at 1 and 3 days postoperatively (scale bar = 100 μm). (H) H&E staining of liver sections (scale bar = 100 μm) and the quantitative analysis of liver necrosis areas. (I) mRNA levels of inflammatory factors in the liver at 1 day. (J) Protein levels of IL6 in the liver. EIF5 was used as the internal control. (K) Plasma ALT levels and (L) plasma MDA levels. (M) Bubble diagram of PE levels in the liver at 1 day postoperatively. Data present the mean ± SEM. ∗*p <* 0.05. Mid-aged, middle-aged. Figure 6C was created with Biorender.com.

Therefore, we hypothesized that PCYT2 inhibition might confer protective effects in pathophysiologic conditions with impaired liver regeneration. MASH and aging are two well-documented conditions associated with exacerbated liver injury following PHx. Thus, we detected the protein level of PCYT2 in MASH and middle-aged mouse livers. We found that its level remained unchanged in FPC diet-induced MASH 19, 25 liver compared to controls (Figure S6A). In contrast, we observed higher protein levels of PCYT2 in the livers of middle-aged mice than in those of young mice (Figure 6A-B). We previously reported that in middle-aged mice, senescent liver cells affected the liver environment in a paracrine manner 27. We treated HepG2 cells with DOX to induce senescence and treated non-senescent HepG2 cells with conditioned medium from senescent hepatocytes (Figure 6C). We observed that conditioned medium from senescent hepatocytes substantially increased PCYT2 in non-senescent cells (Figure 6D). Macrophages and endothelial cells are two non-parenchymal cell types in the liver. We also induced senescence in macrophages (RAW264.7 cells) or endothelial cells (EA.hy926 cells) using DOX and collected the conditioned medium to treat non-senescent hepatocytes. The PCYT2 levels in non-senescent hepatocytes were also increased (Figure S6B-E). These data indicate that the senescence-associated secretory phenotype of senescent cells elevated hepatic PCYT2 levels.

Subsequently, middle-aged mice were pretreated with meclizine, followed by 70% PHx (Figure 6E). Meclizine treatment did not affect liver weight, body weight, and the liver/body weight ratio (Figure S7A-C). Although the PCNA level was increased by meclizine in middle-aged mouse livers 1 day after 70% PHx (Figure 6F), the Ki67-positive cell number was not significantly changed in the liver (Figure 6G). PCNA levels were comparable between the meclizine-treated and control mice 8 days postoperatively (Figure S7D). Notably, H&E staining revealed necrotic foci in middle-aged mouse livers at 1 day after 70% PHx, which was ameliorated by meclizine treatment (Figure 6H). Moreover, meclizine reduced plasma ALT levels and the expressions of inflammatory cytokines (Figure 6I-K). Plasma MDA levels were also decreased following meclizine treatment (Figure 6L). The phospholipidomics showed that the meclizine decreased hepatic PE content (Figure 6M). These data indicate that PCYT2 inhibition by meclizine before 70% PHx attenuate liver injury in middle-aged mice.

### Synthesis of GalNAc-conjugated PEG-PCL-Meclizine NPs

**Figure 7:**
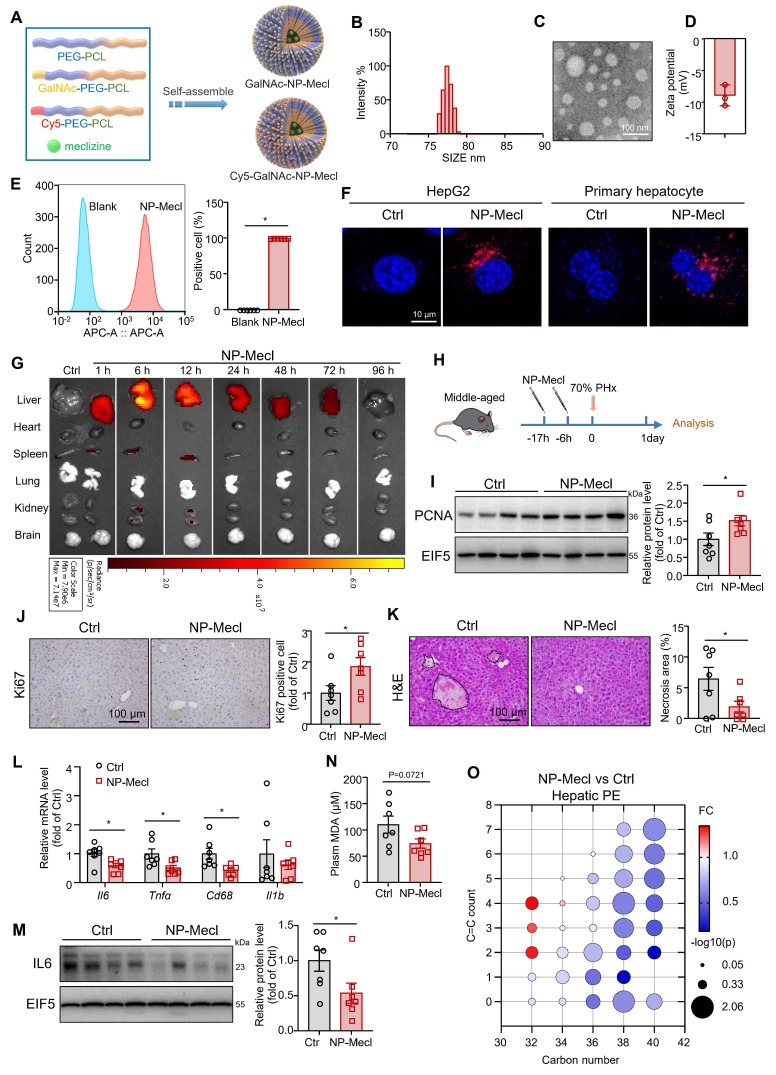
** NP-meclizine promoted normal liver regeneration in middle-aged mice. (A)** Schematic illustration of the procedure used to prepare NP-meclizine. **(B)** The size distribution, **(C)** transmission electron microscope images (scale bar = 100 nm), and **(D)** the zeta potential of NP-meclizine. **(E-F)** HepG2 cells and primary mouse hepatocytes were treated with 3.75 μg/mL Cy5-NP-meclizine for 6 h. (E) Cellular uptake of Cy5-NP-meclizine in HepG2 cells was detected using flow cytometry (n = 6 independent experiments). (F) Representative Confocal images of Cy5-NP-meclizine in HepG2 cells and primary mouse hepatocytes (scale bar = 10 μm). **(G)** Mice were injected with 80 μg Cy5-NP-meclizine. The fluorescence imaging of the liver, heart, spleen, lung, kidney, and brain were obtained at 1, 6, 12, 24, 48, 72, and 96 h after the injection.** (H-O)** Ten-month-old mice were administered 150 μg NP-meclizine (containing 25 μg meclizine) or PBS 17 h and 6 h before 70% PHx, and subsequently the tissues were harvested for analysis at 1 day postoperatively (n = 7 mice per group; H). (I) Protein levels of PCNA in the liver; EIF5 was used as the internal control. (J) Immunohistochemical staining of Ki67 in the liver (scale bar = 100 μm). (K) H&E staining of liver sections (scale bar = 100 μm) and the quantitative analysis of liver necrosis areas. (L) mRNA levels of inflammatory genes in the liver. (M) Protein levels of IL-6 in the liver; EIF5 was used as the internal control. (N) Plasma MDA level. (O) Bubble diagram of PE levels in the liver. Data present the mean ± SEM. ∗*p <* 0.05. NP-Mecl, NP-meclizine.

Meclizine is also well known as a blocker of the histamine H1 receptor, a G protein-coupled receptor 47. To further exclude its effects on membrane receptors, we employed NP to deliver meclizine into cells, which enabled better targeting of intracellular targets. Additionally, NP-based drug delivery systems can provide better targeting to the liver, which is beneficial for avoiding side effects and enhancing therapeutic efficiency. The chimeric polymer, PEG-PCL, was used to encapsulate meclizine. GalNAc specifically binds to the salivary acid glycoprotein receptor on hepatocyte membranes 31, 32, 48. Therefore, the micelles were conjugated with GalNAc to target the liver (NP-meclizine; Figure 7A and Figure S8). NP-meclizine were mainly distributed with a hydrodynamic diameter of 70-100 nm (Figure 7B-C) and a negative zeta potential (Figure 7D). NP-meclizine was labeled with Cy5 to visualize the cellular uptake and *in vivo* distribution of the drug-loaded NP. Flow cytometry and confocal microscopy revealed that hepatocytes internalized NP-meclizine (Figure 7E-F). Subsequently, Cy5-labeled NP-meclizine was intravenously administered to mice via the tail vein. We found NP-meclizine was selectively enriched in the liver (Figure 7G). Moreover, NP-meclizine accumulation in the liver was observed 1 h after injection and lasted for 48 h, with the highest level occurring at 6-12 h (Figure 7G).

### NP-meclizine facilitated liver regeneration in middle-aged mice

Then, we pretreated middle-aged mice with 150 μg of NP-meclizine (containing 25 μg of meclizine) at 17 h and 6 h before 70% PHx (Figure 7H). Similar to meclizine, NP-meclizine increased the PCNA level and the number of Ki67-positive cells in the liver (Figure 7I-J), suggesting an enhanced proliferation signal. However, liver weight and liver-to-body weight ratio remained unchanged (Figure S9A-C). NP-meclizine reduced the number of necrotic foci in the livers of middle-aged mice after 70% PHx (Figure 7K). The mRNA levels of *Il6*, *Tnfα*, and *Cd68* were lower in the NP-meclizine-treated mice than in the control mice, indicating better inflammatory resolution (Figure 7L). NP-meclizine also decreased the protein levels of IL6 (Figure 7M). Similarly, the plasma MDA levels tended to decrease (*p* = 0.0721; Figure 7N). However, the ALT levels were comparable between the two groups (Figure S9D). We noted that NP-meclizine reduced the hepatic levels of PE species (Figure 7O). Moreover, the dose of meclizine delivered via NP was lower than that administered systemically.

**Figure 8:**
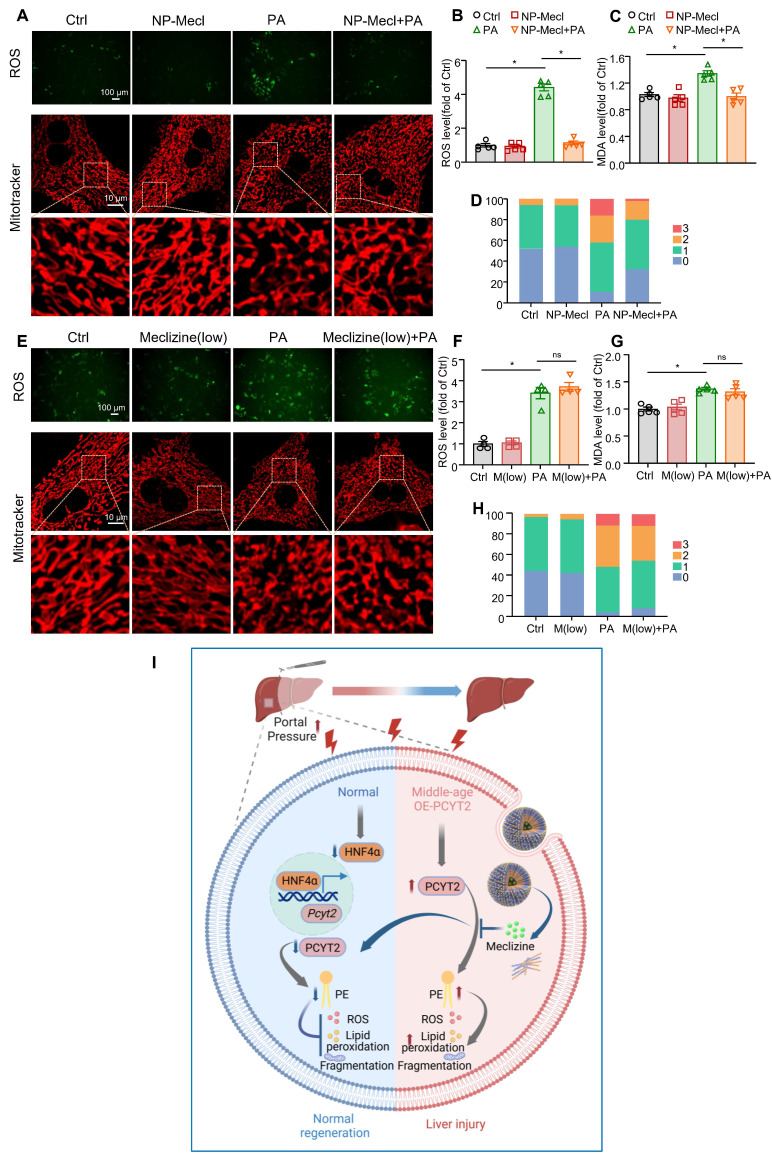
** The effects of NP-meclizine on ROS and MDA levels in fatty acid-overloaded hepatocytes (A-D).** Primary mouse hepatocytes were treated with 3.75 μg/mL NP-meclizine (containing 1 μM meclizine) for 17 h, then the cells were co-treated with PA (100 μM) for another 24 h. **(E-H)** Primary mouse hepatocytes were treated with meclizine (1 μM; the same dose containing in 3.75 μg/mL NP-meclizine) for 17 h; subsequently, the cells were co-treated with or without PA (100 μM) for another 24 h. Representative images of ROS in
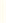
cells (upper panel of A, E; scale bar = 100 μm) and the quantitative analysis of ROS (B, F); the MDA content in hepatocytes (C, G); (lower panel of A, E; scale bar = 10 μm) mitochondrial morphology was evaluated via MitoTracker. (D, H) Quantitative scoring of mitochondrial morphology in 50 hepatocytes from three biologically independent experiments. n = 3**-**5 independent experiments. Data are presented as the mean ± SEM. ∗*p <* 0.05. NP-Mecl, NP-meclizine; M, meclizine; PA, palmitate. **(I)** Model of how PE and PCYT2 participate in 70% PHx-induced liver regeneration. Following 70% PHx, increased portal pressure downregulated PCYT2 through HNF4α. Decreased PCYT2 level and PE content defense hepatocytes from injury during this process, which is important for normal liver regeneration. PCYT2 overexpression exacerbated liver injury after 70% PHx. Middle-aged mouse livers showed higher PCYT2 levels. Inhibition of PCYT2 with NP-meclizine attenuated liver injury in middle-aged mice after 70% PHx. The illustration was created with Biorender.com.

The hepatocytes were treated with NP-meclizine at a dose of 3.75 μg/mL. NP-meclizine treatment reduced PA-induced elevations in ROS and MDA levels, as well as mitochondrial fragmentation (Figure 8A-D). The meclizine dose in NP-meclizine used to treat hepatocytes was lower than that used to treat hepatocytes, as shown in Figure 5. However, the carrier-free meclizine at a dose equal to that contained in NP-meclizine (1 μM) did not alter ROS or MDA levels, nor did it affect mitochondrial fragmentation in PA-treated hepatocytes (Figure 8E-H). Additionally, the beneficial effects of NP-meclizine on reducing ROS were blunted by PE (Figure S10A). We knocked down HNF4α in hepatocytes before they were treated with NP-meclizine and PA. We found that in HNF4α-knockdown cells, NP-meclizine did not further reduce ROS levels (Figure S10B). These data indicated that NP-meclizine enhanced the therapeutic efficacy of meclizine. Therefore, NP-meclizine, designed to bypass the H1 receptor, ameliorated liver injury after 70% PHx in middle-aged mice.

Excess ROS production is also important for APAP-induced liver injury. We further found that NP-meclinzine also attenuated APAP-induced liver injury, as evidenced by reduced necrotic area in the liver and plasma ALT levels (Figure S11A-B). The expression of inflammatory factors, including *Il6*, *Tnfa,* and *Il1b,* were also decreased by NP-meclizine (Figure S11C).

## Discussion

In this study, we performed targeted phospholipidomics to explore the changes in phospholipid levels at different time points after 70% PHx. The phospholipid components changed substantially at 1, 3, and 5 days postoperatively and were comparable between the PHx and sham groups 8 days after surgery. Among these changes, decreased PE content at 1 day after PHx was a feature of the phospholipid change caused by the surgery. We also found that hepatic PCYT2 levels greatly decreased in the early stages of liver regeneration.

Regarding the mechanism by which PCYT2 expression is downregulated, we identified it as a target gene of *HNF4α*. The expression level of HNF4α was inhibited at the early phase of liver regeneration after PHx 38. Higher HNF4α levels are involved in impaired liver regeneration in aged mice 49. After 70% of the liver tissue is removed, portal pressure is immediately elevated because of the smaller vascular bed in the liver remnant 50. In this study, we found that HNF4α and PCYT2 levels were suppressed by increased portal pressure with an *ex vivo* liver perfusion system. The *ex vivo* liver perfusion system enables better control of chemical signals and preserves the hemodynamic characteristics of the liver. Thus, our results suggest that mechanical signals can regulate phospholipid remodeling.

Although PCYT2 overexpression decreased PCNA expression, it did not affect liver weight. In contrast, PCYT2 overexpression aggravated liver injury induced by 70% PHx. Several studies have consistently highlighted defense mechanisms against liver injury induced by PHx. Mitochondrial calcium uptake 1 deficiency-induced Ca^2+^ overload causes extensive liver damage after 70% PHx without affecting the early priming of liver regeneration 41. Additionally, loss of β1-integrin in hepatocytes leads to severe liver necrosis after PHx 51. The P2X4 purinergic receptor also protects hepatocytes from injury after 70% PHx 4. In this study, we found that decreased PCYT2 expression protected the liver during the early phase of liver regeneration after 70% PHx.

Higher PE or PCYT2 levels are reportedly associated with tissue injury. PEMT knockout decreases the hepatic PC/PE ratio in non-alcoholic fatty liver disease mice and decreases their survival rates after PHx 52, suggesting that high PE levels impair liver regeneration. Increased PE and PCYT2 expressions were associated with more severe kidney damage induced by hyperhomocysteinemia in a 2-kidney, 1-clip mouse model 53. PCYT2 downregulation may be involved in helenalin-mediated hepatoprotective effects in CCl_4_-treated mouse livers 54. PEMT deficiency inhibited the conversion of PE to PC and activated mitochondrial respiration 55. Elevated PE levels are associated with increased mitochondrial respiration and fragmentation 56. PCYT2 inhibitor meclizine shifts energy metabolism from mitochondrial respiration to glycolysis 43, which may decrease mitochondrial ROS production 57. Therefore, this study implies that the decreased PCYT2 expression and PE content temporarily suppress mitochondrial respiration to decrease ROS production during the early phase of liver regeneration after 70% PHx. Additionally, increased PE levels are closely related to lipid peroxidation 18, which leads to cell injury. Thus, decreased PE and PCYT2 levels in the early stages of liver regeneration constitute a defense strategy against cell injury caused by PHx-induced stress signals.

PCYT2 deficiency in hepatocytes increases TG synthesis 58. This is because PE synthesis requires diacylglycerol, which can also be converted to TG 58. TG accumulation is a hallmark of the liver during the early stages of PHx-induced regeneration. However, PCYT2 overexpression did not alter TG accumulation after treatment with 70% PHx.

Pcyt2 expression is upregulated in bone marrow mesenchymal stem cells from old rats compared to those from young rats 59. Sirt1 protects against age-related diseases 60, and Pcyt2 expression is upregulated in the liver of hepatocyte-specific Sirt1 null mice 61. We found that PCYT2 levels were elevated in the liver of middle-aged mice. More apoptotic cells have been reported in the livers of older mice than in those of young mice after PHx 7, 8. Plasma ALT and aspartate transaminase levels are also increased in aged or middle-aged mice 1-3 days after PHx 7, 8, 49. Parameters of oxidative stress are higher in the livers of old rats than in those of young rats after PHx 62. Clinically, advanced age is an independent risk factor for severe complications after major hepatectomy with bile duct resection 63. Therefore, middle-aged mice were pretreated with meclizine to inhibit PCYT2 expression. We found that meclizine protects against PHx-induced liver injury in middle-aged mice.

Meclizine is proved as a PCYT2 inhibitor 42. However, it is also a well-known human histamine H1 receptor blocker, which is a G protein-coupled receptor 47. Thus, the systemic administration of meclizine also targets its membrane receptors and may affect other organs. Therefore, we developed NPs for the delivery of meclizine. The NPs are taken up by cells, preventing meclizine from acting on its membrane receptors. Moreover, the enrichment of NP-meclizine in the liver enabled better organ targeting. We also found that NP-meclizine attenuated liver injury in middle-aged mice subjected to 70% PHx. Additionally, the dose of meclizine administered via the NP-meclizine system was lower than that used for systemic delivery. These data indicate that enrichment in the liver also increased the therapeutic efficacy of meclizine.

In addition to the current focus on PE, we also found that other phospholipids were changed during this process. For 1 day after 70% PHx, two ether PCs were significantly increased, warranting further exploration. The mechanisms underlying senescence-associated secretory phenotype-mediated elevation of PCYT2 also require further investigation.

In summary, the results of this study focused on phospholipid remodeling during liver regeneration after 70% PHx. We detected a decrease in the PE content and downregulation of PCYT2, a rate-limiting enzyme in PE biogenesis, during the early phase of PHx-induced liver regeneration. PCYT2 is a target gene of *HNF4α*, which was decreased by 70% PHx-increased portal pressure. PCYT2 downregulation protects against liver injury and promotes normal liver regeneration. PCYT2 inhibition with NP-meclizine attenuated liver injury in middle-aged male mice after exposure to 70% PHx (Figure 8I).

## Supplementary Material

Supplementary figures and table.

## Abbreviations

AAV: adeno-associated virus
ALT: alanine aminotransferase
APAP: acetaminophen
DOX: doxorubicin
FBS: fetal bovine serum
GalNAc: N-acetylgalactosamine
HNF4α: hepatocyte nuclear factor 4 Alpha
H&E: hematoxylin-eosin
LC-MS/MS: liquid chromatography-tandem mass spectrometry
MDA: malondialdehyde
NP: Nano-particle
PA: palmitate
PC: Phosphatidylcholine
PCL: Polycaprolactone
PCNA: proliferating cell nuclear antigen
PCYT2: Phosphate cytidylyltransferase 2, ethanolamine
PE: Phosphatidylethanolamine
PEG: Polyethylene glycol
PEMT: phosphatidylethanolamine N-methyltransferase
PHx: Partial Hepatectomy
ROS: Reactive Oxygen Species
