# Supplementary material for: Downregulation of PCYT2 by increased portal pressure safeguards liver regeneration after partial hepatectomy
Source: Theranostics. 2026 Jan 1;16(2):810–29. doi: 10.7150/thno.118755 (PMC12674937; doi:10.7150/thno.118755)
Supplement: Supplementary file 1 — Supplementary figures and table. [file thnov16p0810s1.pdf]

Figure S1

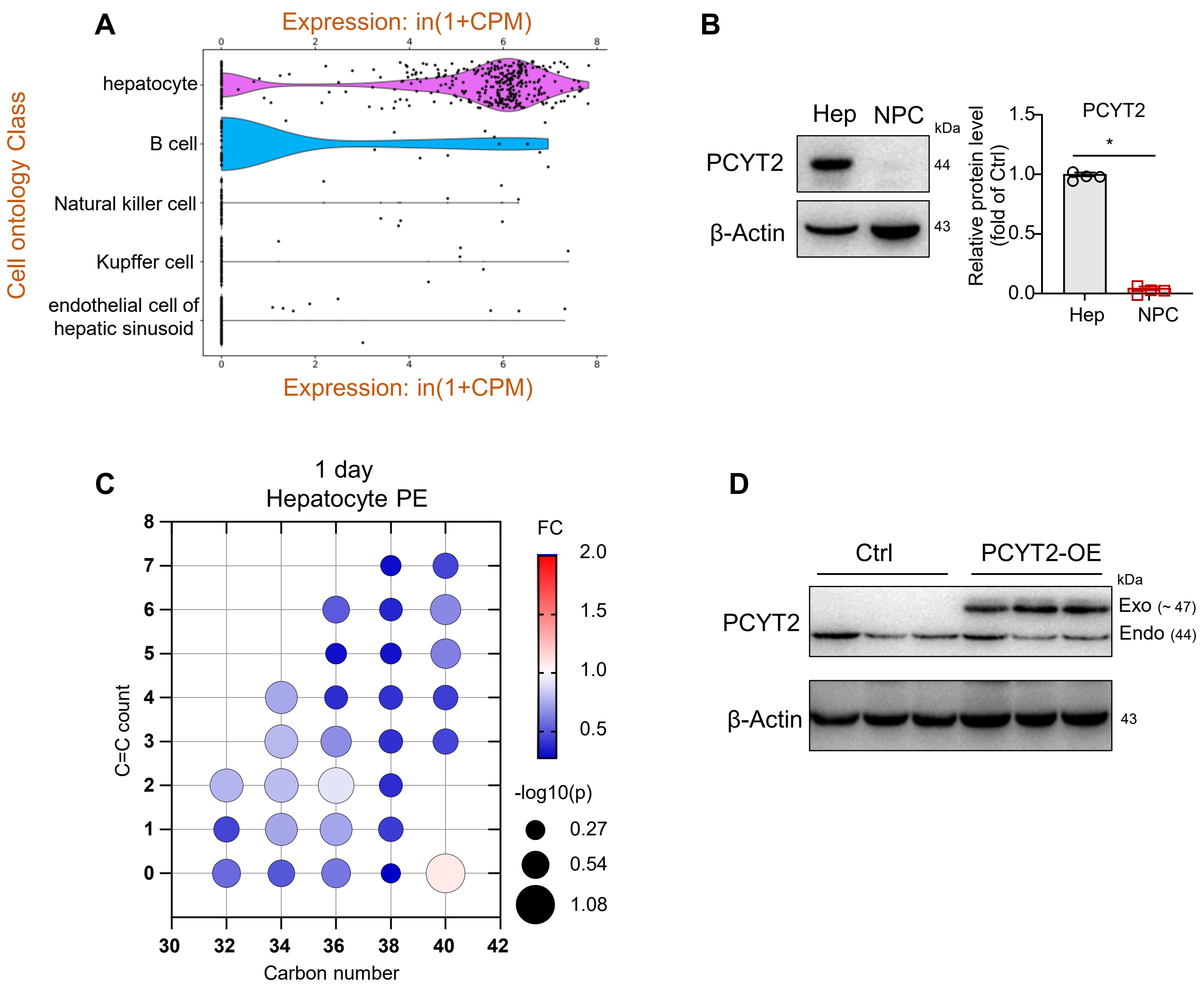

Figure S1. *Pcyt2* is expressed mainly in hepatocytes in the mouse liver and was overexpressed by PCYT2-expressing AAVs. (A) The single-cell sequencing database Tabula Muris shows the expression pattern of Pcyt2 in diverse liver cells. **(B)** Hepatocytes and non-parenchymal cells (NPC) were isolated from mouse livers. Western blot analysis of the protein levels of PCYT2. n = 4 mice per group. **(C)** Bubble diagram of PE levels in isolated hepatocytes at 1 day after surgery. n = 8 mice per group. **(D)** Eight-week-old mice were administered AAV-Pcyt2-flags bearing the TBG promoter or control AAV through tail vein injection. Western blot analysis of the protein levels of PCYT2 in the liver. β-Actin was used as the internal control. n = 6 mice per group. Data present the mean ± SEM. $*p < 0.05$. Exo, exogenous; Endo, endogenous.

Figure S2

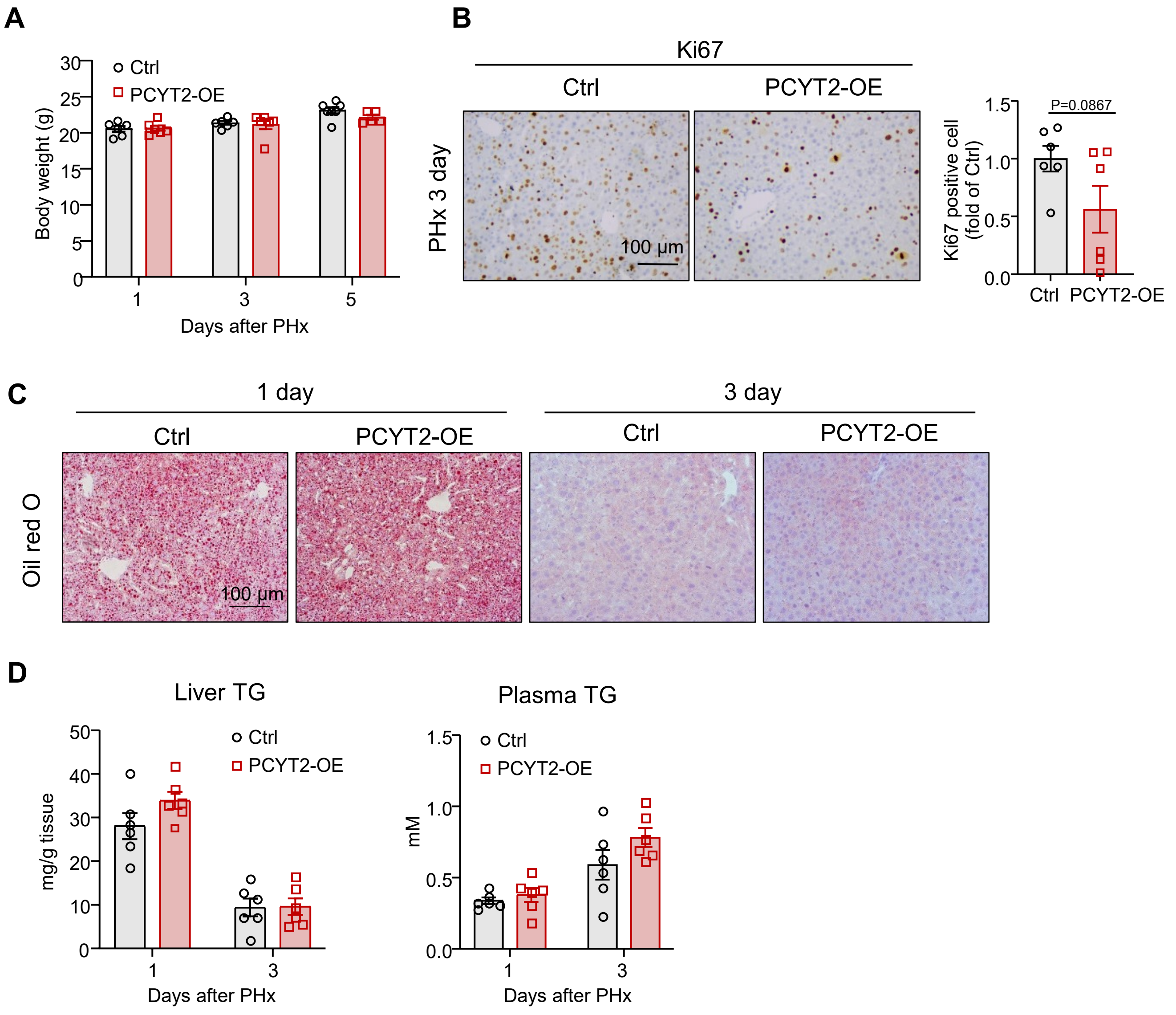

Figure S2. Effects of PCYT2 overexpression on Ki67-positive cell counts and lipid accumulation in the liver after 70% partial hepatectomy (PHx). Eight-week-old mice were administered AAV-PCYT2-flags bearing the TBG promoter or control AAV through tail vein injection. After 10 days, the mice were subjected to 70% PHx, and the tissues were harvested for analysis at the indicated time points. **(A)** Body weights of the mice. **(B)** Immunohistochemical staining of Ki67 in liver sections from mice at 3 days (scale bar = 100 µm). **(C)** Oil red O staining of the liver (scale bar = 100 µm). (**D)** Plasma and liver triglyceride (TG) contents. n = 5–7 mice per group. Data present the mean ± SEM. ∗p < 0.05. TG, triglyceride.

Figure S3

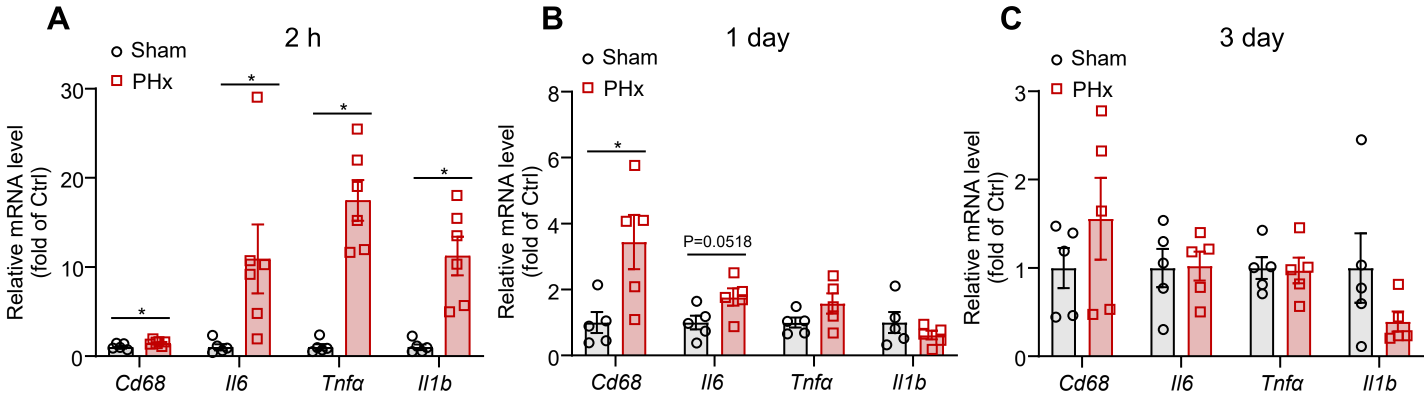

Figure S3. **The expression of inflammatory factors in the liver after partial hepatectomy (PHx).** Eight-week-old mice were subjected to 70% PHx or sham surgery. qPCR analysis of the mRNA levels of inflammatory factors in mouse livers at 2 h (A), 1 day (B) or 3 days postoperatively (C). n = 5–6 mice per group. Data present the mean ± SEM. $*p < 0.05$.

Figure S4

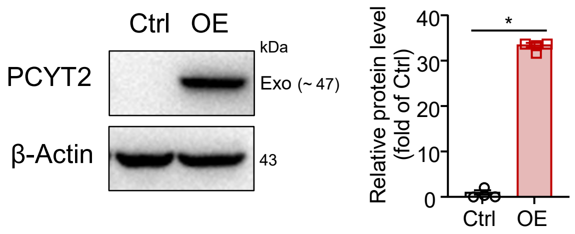

Figure S4. **PCYT2 was overexpressed in primary mouse hepatocytes via adenovirus.** Primary mouse hepatocytes were treated with Ad-PCYT2 or Ad-Ctrl for 48 h. Western blot analysis of the protein levels of PCYT2. β-Actin was used as the internal control. n = 4 independent experiments. Data present the mean ± SEM. $*p < 0.05$. OE, PCYT2 overexpression. Exo, exogenous.

Figure S5

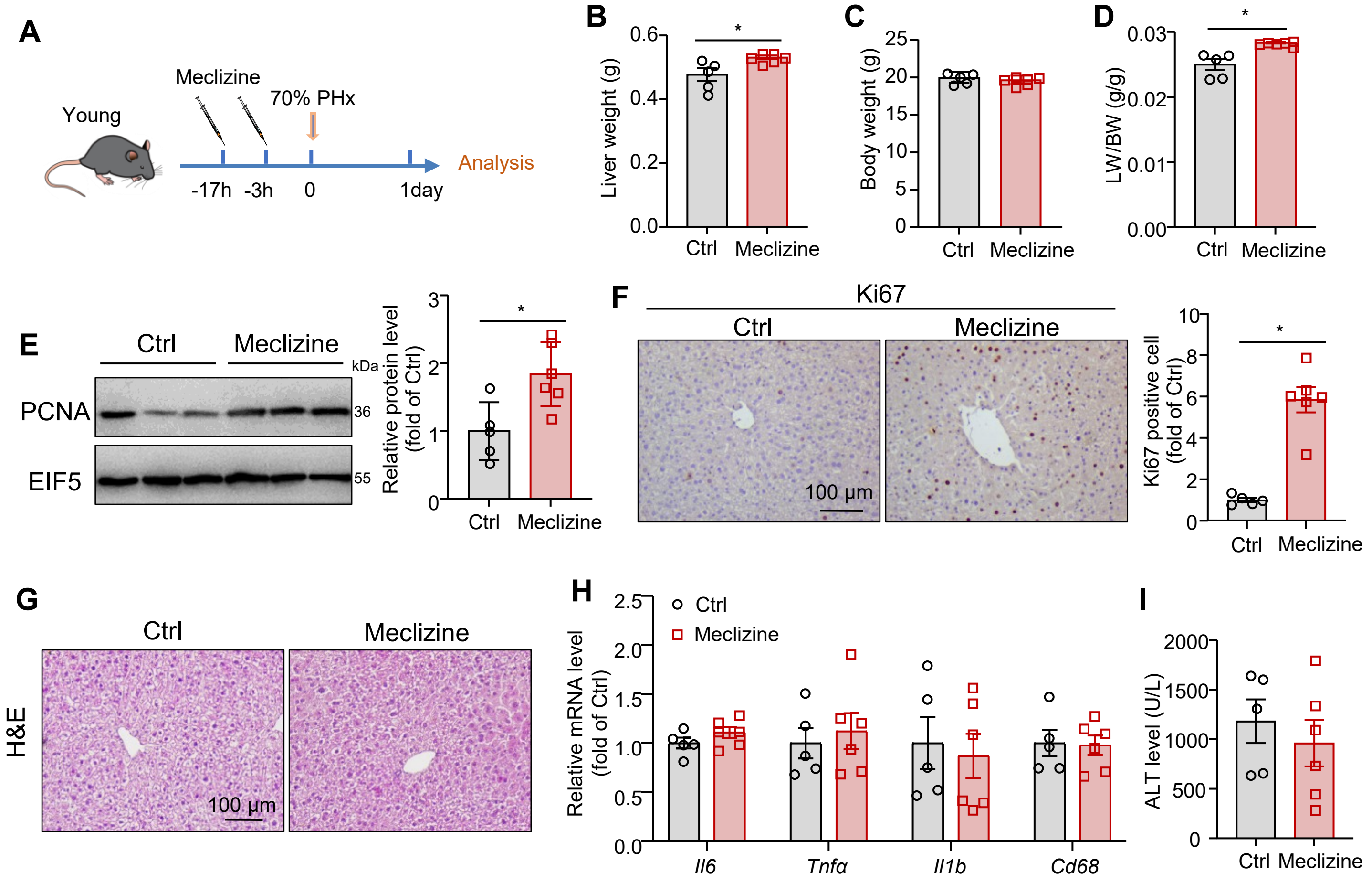

Figure S5. Inhibition of PCYT2 by meclizine promoted liver regeneration in eight-week-old mice. Eight-week-old mice were administered 60 mg/kg meclizine or vehicle 17 h and 3 h before 70% PHx, and then the tissues were harvested for analysis at 1 day after surgery **(A)**. **(B)** Liver weights, **(C)** body weights, **(D)** and liver-to-body weight ratios of the mice. **(E)** Western blot analysis of the protein levels of PCNA in the liver. EIF5 was used as the internal control. **(F)** Immunohistochemical staining of Ki67 in liver sections (scale bar = 100 μm) and quantification of Ki67-positive cells. **(G)** H&E staining of liver sections (scale bar = 100 μm). **(H)** qPCR analysis of the mRNA levels of inflammatory factors in the liver. **(I)** Plasma ALT levels. n = 5 in the control group, n = 6 in the meclizine group. Data present the mean ± SEM. $*p < 0.05$. LW/BW, liver-to-body weight ratios.

Figure S6

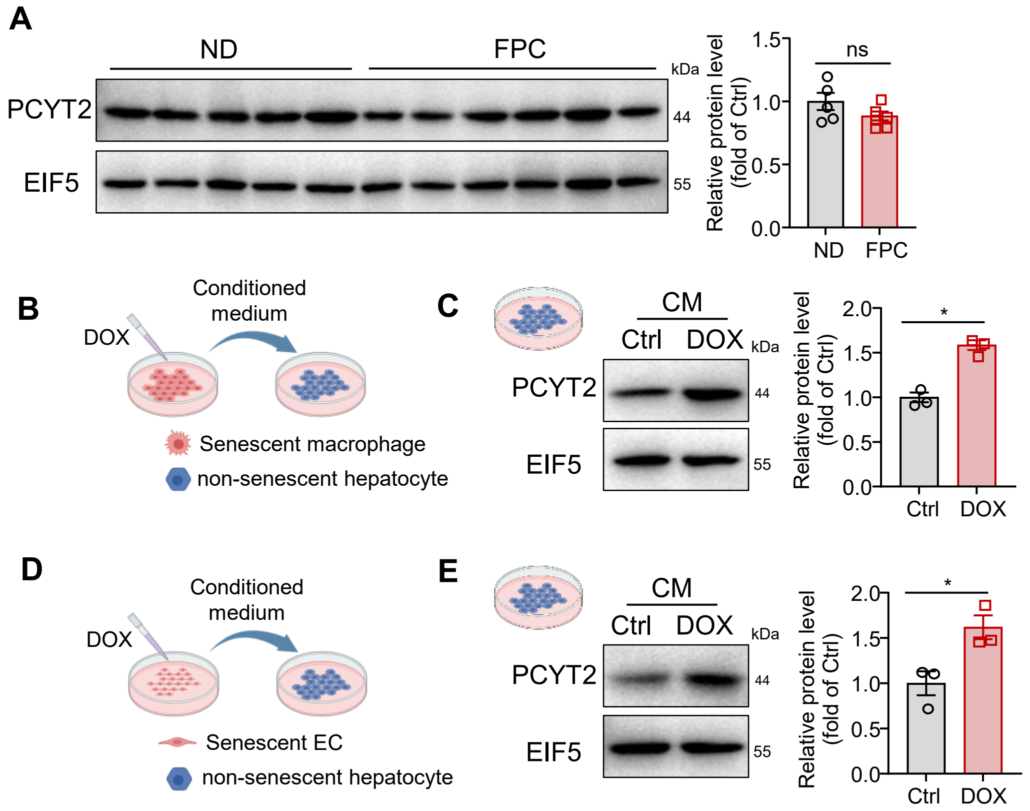

Figure S6. The protein level of PCYT2 in MASH mouse livers and in hepatocytes treated with conditioned medium from senescent cells. (A) Eight-week-old mice were fed a diet rich in fructose, palmitate, and cholesterol (FPC) or a normal diet (ND) for 24 weeks. Western blot analysis of the protein levels of PCYT2. EIF5 was used as the internal control. n = 5–6 mice per group. **(B–C)** Macrophages (RAW264.7 cells) were treated with doxorubicin (DOX) to induce senescence. The conditioned medium was collected to treat non-senescent HepG2 cells. The protein levels of PCYT2 in non-senescent hepatocytes treated with the conditioned medium; EIF5 was used as the internal control. **(D–E)** Endothelial cells (EA.hy926 cells) were treated with DOX to induce senescence. The conditioned medium was collected to treat non-senescent HepG2 cells. The protein levels of PCYT2 in non-senescent hepatocytes treated with conditioned medium; EIF5 was used as the internal control. (C and E) n = 3 independent experiments. Data present the mean ± SEM. ∗$p < 0.05$. EC, Endothelial cells.

Figure S7

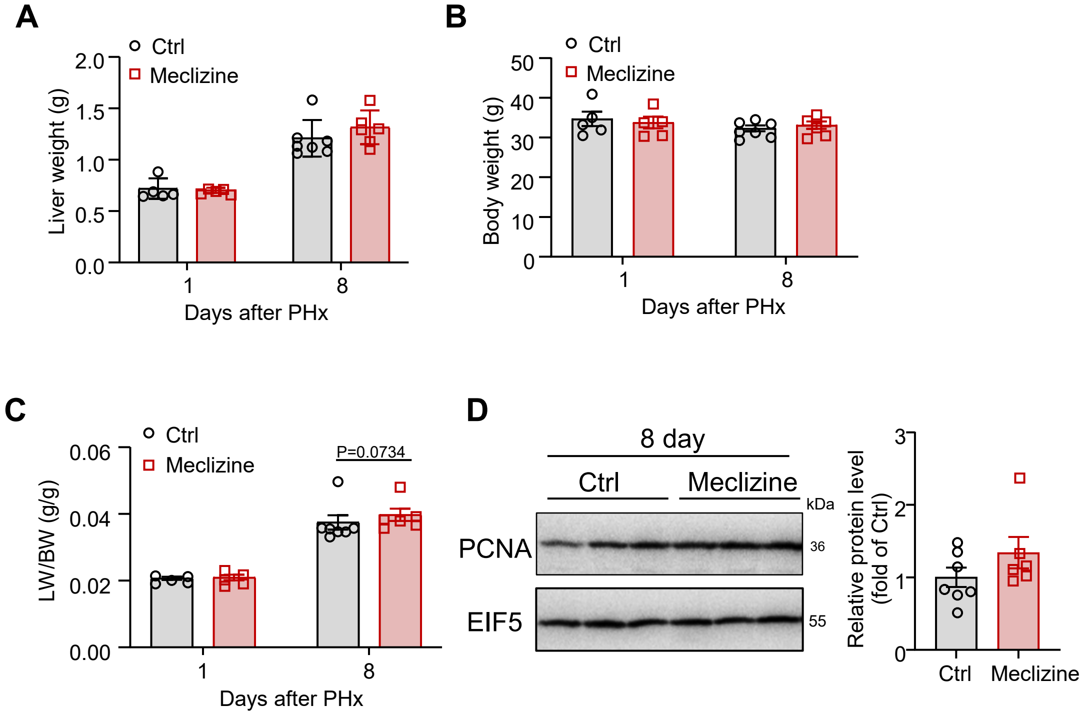

Figure S7. Liver weights of meclizine-treated middle-aged mice after 70% partial hepatectomy (PHx). Ten-month-old mice were administered 60 mg/kg meclizine or vehicle 17 h and 3 h before 70% PHx, and then the tissues were harvested for analysis at 1 day (n = 5 mice per group) or 8 days (n = 7 mice in the control group; n = 6 mice in the meclizine group) postoperatively. **(A)** Liver weights, **(B)** body weights, **(C)** and liver-to-body weight ratios of the mice. **(D)** Western blot analysis of the protein levels of PCNA in mouse livers at 8 days after surgery. EIF5 was used as the internal control. Data present the mean ± SEM. $*p < 0.05$. LW/BW, liver-to-body weight ratios.

Figure S8

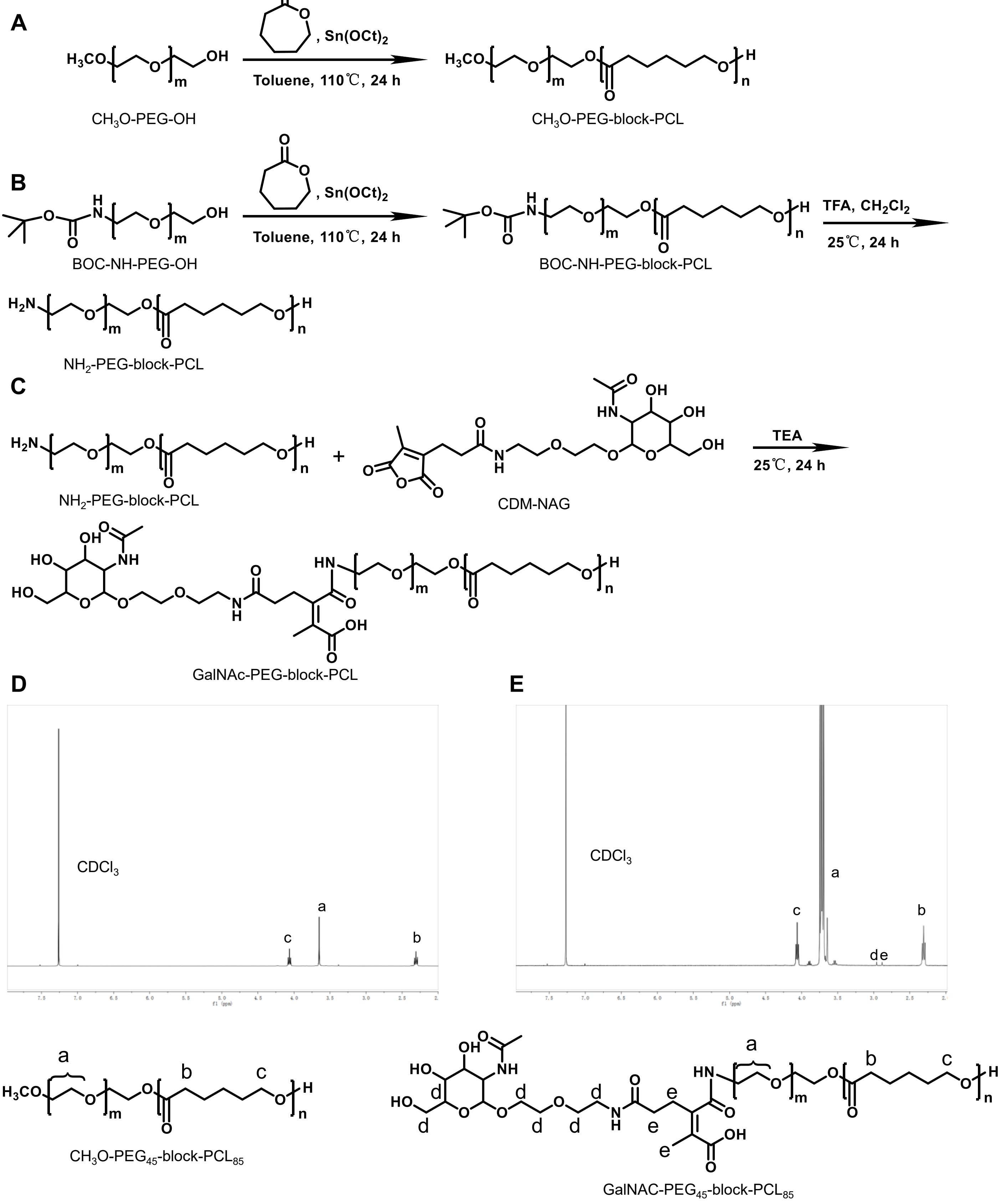

Figure S8. Characterization of the CH3O-PEG-block-PCL copolymer and GalNAc-PEG-block-PCL copolymer. Synthesis routes for the (A) CH3O-PEG-block-PCL copolymer and (B, C) GalNAc-PEG-block-PCL copolymer. (D) 1H-NMR spectrum of the CH3O-PEG-block-PCL copolymer. (E) 1H-NMR spectrum of the GalNAc-PEG-block-PCL copolymer.

Figure S9

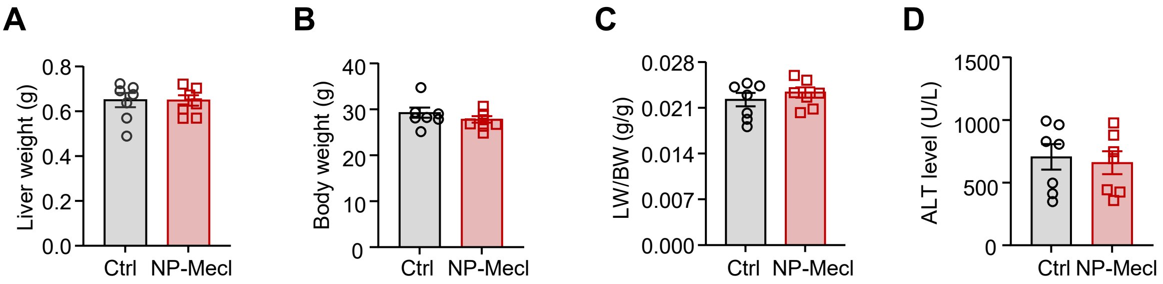

Figure S9. Liver weights of NP-meclizine-treated middle-aged mice after 70% partial hepatectomy (PHx). Ten-month-old mice were administered 150 μg NP-meclizine (containing 25 μg meclizine) or PBS 17 h and 6 h before 70% PHx, and then the tissues were harvested for analysis 1 day postoperatively (n = 7 mice per group). (**A**) Liver weights, (**B**) body weights, (**C**) and liver-to-body weight ratios. (**D**) Plasma ALT levels. Data present the mean ± SEM. $*p < 0.05$. LW/BW, liver-to-body weight ratios; NP-Mecl, NP-meclizine.

Figure S10

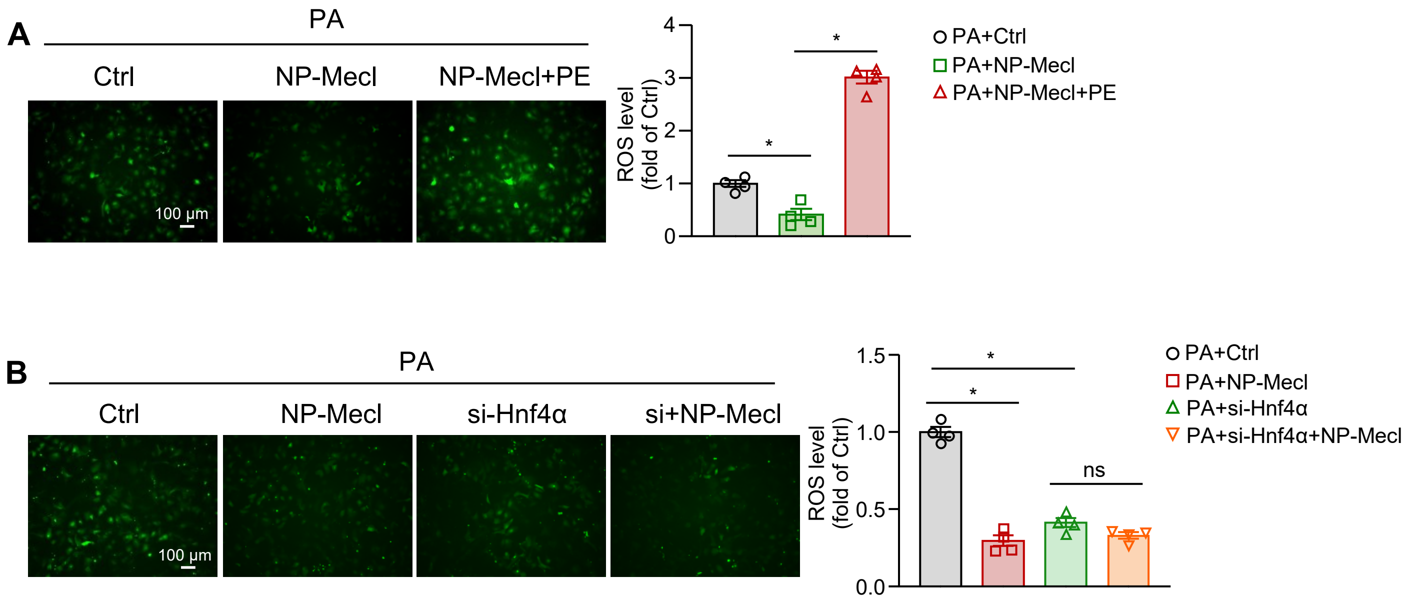

Figure S10. Influence of PE or HNF4α knockdown on the NP-meclizine-mediated ROS reduction in fatty acid-overloaded hepatocytes. (A) Primary mouse hepatocytes were treated with 3.75 μg/mL NP-meclizine (containing 1 μM meclizine) for 17 h, and then the hepatocytes were co-treated with PA (100 μM) and PE (10 μM) for another 24 h. **(B)** Primary mouse hepatocytes were transfected with si-Hnf4α or si-NC; 24 h later, the hepatocytes were treated with 3.75 μg/mL NP-meclizine (containing 1 μM meclizine) for 17 h, and then the hepatocytes were co-treated with PA (100 μM) for another 24 h. (A and B) Representative images and quantitative analysis of ROS in hepatocytes (scale bar = 100 μm). n = 4 independent experiments. Data present the mean ± SEM. $*p < 0.05$. NP-Mecl, NP-meclizine.

Figure S11

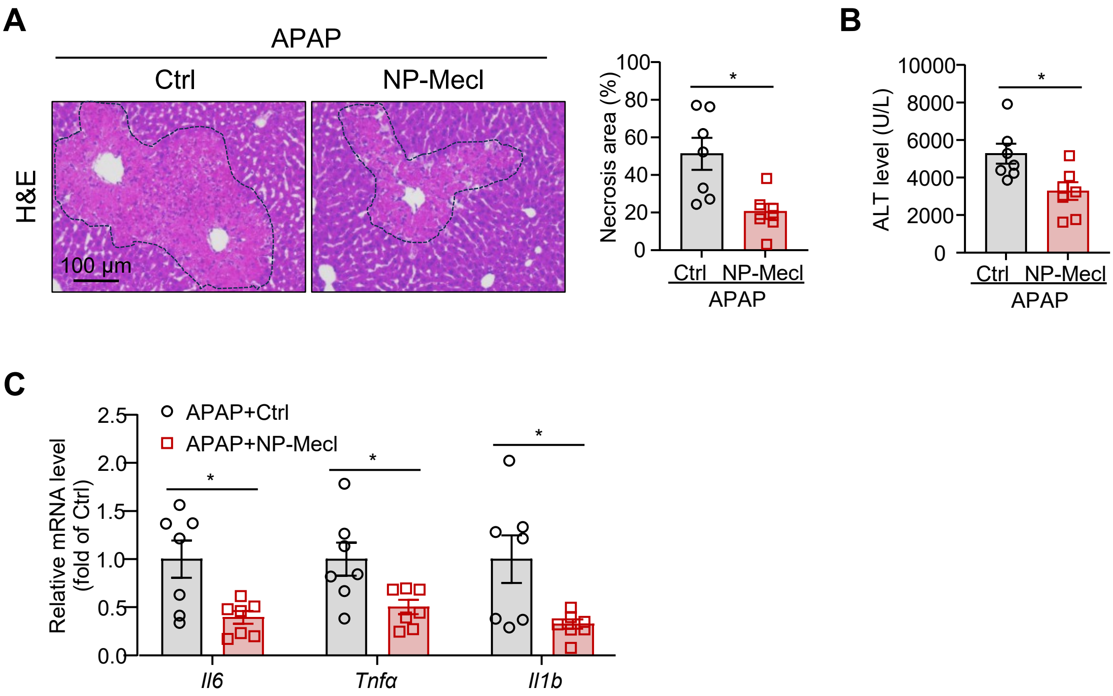

Figure S11. NP-meclizine attenuated acetaminophen (APAP)-induced liver injury. Eight-week-old mice were administered 180 μg of NP-meclizine (containing 25 μg of meclizine) or PBS 17 h and 6 h before being treated with APAP for 24 h. **(A)** H&E staining of liver sections and the quantitative analysis of liver necrosis areas (scale bar = 100 μm). **(B)** Plasma ALT levels. **(C)** qPCR analysis of the mRNA levels of inflammatory factors in mouse livers. n = 7 mice in the control group. Data present the mean ± SEM. $*p < 0.05$. NP-Mecl, NP-meclizine.

Table S1. List of oligonucleotide primer pairs used in qPCR

Target gene	Sense primer	Antisense primer
18S (h/m)	5'-GGAAGGGCACCACCAGGAGT-3'	5'-TGCAGCCCCGGACATCTAAG-3'
Pcyt2 (m)	5'-AGGCTGGGAGGTACAGAGAG-3'	5'-ATCTCCTGGCTGCTGTGATG-3'
Pemt (m)	5'-CTGTGGAGGCTTCGGCAATA-3'	5'-AACCTAGGAATGCAAGGCCC-3'
Cept (m)	5'-CAGCTTCTCTCAGGAACCGT-3'	5'-GCCACAGTACAAAGTGCAGG-3'
Pisd (m)	5'-CTAGTCACTGGGAGGTGTCC-3'	5'-AGCTTACGCCGGAAGAACTC-3'
Etnk1 (m)	5'-GTCAATCAGTTTGCGTTGGCT-3'	5'-CCGCATACCCAAGGAAGTCG-3'
Hnf4α (m)	5'-GGGGTTCCTGCAGATCACAT-3'	5'-TCCCAGAGATGGGAGAGGTG-3'
Il6 (m)	5'-CACTTCACAAGTCGGAGGCT-3'	5'-GCCACTCCTTCTGTGACTCC-3'
Il1b (m)	5'-CTCTGTGACTCATGGGATG-3'	5'-CTTCTTCTTTGGGTATTGC-3'
Tnfα (m)	5'-CTGTGAAGGGAATGGGTGTT-3'	5'-CAGGGAAGAATCTGGAAAGGTC-3'
Cd68 (m)	5'-CCCAAGGAACAGAGGAAG-3'	5'-GTGGCAGGGTTATGAGTG-3'
Hnf4α (h)	5'-TGCGACTCTCCAAAACCCTC-3'	5'-ATTGCCCATCGTCAACACCT-3'

m: *Mus musculus; h: Homo sapiens*
